# Reprogrammable and reconfigurable mechanical computing metastructures with stable and high-density memory

**DOI:** 10.1126/sciadv.ado6476

**Published:** 2024-06-26

**Authors:** Yanbin Li, Shuangyue Yu, Haitao Qing, Yaoye Hong, Yao Zhao, Fangjie Qi, Hao Su, Jie Yin

**Affiliations:** Department of Mechanical and Aerospace Engineering, North Carolina State University, Raleigh, NC, 27606, USA.

## Abstract

Mechanical computing encodes information in deformed states of mechanical systems, such as multistable structures. However, achieving stable mechanical memory in most multistable systems remains challenging and often limited to binary information. Here, we report leveraging coupling kinematic bifurcation in rigid cube–based mechanisms with elasticity to create transformable, multistable mechanical computing metastructures with stable, high-density mechanical memory. Simply stretching the planar metastructure forms a multistable corrugated platform. It allows for independent mechanical or magnetic actuation of individual bistable element, serving as pop-up voxels for display or binary units for various tasks such as information writing, erasing, reading, encryption, and mechanologic computing. Releasing the pre-stretched strain stabilizes the prescribed information, resistant to external mechanical or magnetic perturbations, whereas re-stretching enables editable mechanical memory, akin to selective zones or disk formatting for information erasure and rewriting. Moreover, the platform can be reprogrammed and transformed into a multilayer configuration to achieve high-density memory.

## Teaser

Reconfigurable and multistable metastructures enable reprogrammable mechanical computing with stable and high-density memory.

## INTRODUCTION

Mechanical computing, which uses mechanical components for processing information, boasts a longer history than traditional electronic computing. Ancient mechanical computers date back to the 19th century, often composed of gears and levers for counting, calculation, and display, albeit bulky (*1*). Recently, unconventional mechanical computing has emerged as a strategy for information processing and storage by using novel mechanical systems to augment the traditional electronic computing (*1*). Both the structural forms and constituent materials of mechanical computers have undergone significant transformations, shifting from bulky, rigid mechanism–based gears and levers to intricate deformable structures (see tables S1 and S2 for detailed comparisons and summaries). These structures encompass a spectrum from one-dimensional (1D) beams and trusses to 2D lattices and plates, and to 3D shells and architected materials. They are constructed from various stimuli-responsive and nonresponsive materials, enabling interaction with and adaptation to environments that are not achievable by electronic computing (*1*).

Different from electronic systems, information in mechanical systems can be encoded in deformed patterns, unique constituent material properties (*2*–*7*), and/or structural forms (*8*–*19*). Recent advancements in novel mechanical systems, in particular multistable systems such as mechanical metamaterials (*8*, *20*–*28*), origami/kirigami structures (*3*, *29*–*31*), and mechanical mechanisms (*9*, *32*–*34*), offer unconventional platforms for mechanical memory storage (*23*, *31*), information interaction, encryption (*24*), and mechanical computation (*10*, *17*, *21*, *26*, *29*). A bistable mechanical unit, for instance, exhibits two stable states representing mechanical binary digits (bits), “0” or “1,” for information reading and storage. Basic structural forms of bistable units include constrained beams, curved plates, dome shells, origami/kirigami structures, and balloons (*35*). Periodic tessellation of these bistable units in 1D (*3*, *8*, *30*), 2D (*20*, *21*, *23*, *24*), and 3D (*36*, *37*) configurations results in a multistable mechanical computing system with exponentially increased stable states. This expansion provides vast space for processing information bits.

Recent studies on multistable structures have shown promise in storing and processing binary information, yet substantial challenges persist in achieving functionalities analogous to electronic systems in mechanical computing. First, one major challenge lies in balancing the ease of editing information with the stable storage of data within bistable units (*4*, *17*, *23*, *30*). On the one hand, binary information in mechanical systems should be easily manipulated by reversibly switching and snapping between two stable states. This corresponds to writing and erasing information in response to mechanical forces or external stimuli such as heat, light, electricity, moisture, and magnetic field. Upon removal of actuations, the bits structures stay in their stable states to retain the information without additional energy input. On the other hand, once written or erased, stable memory requires the binary states not be easily interrupted (changed or switched to another state) regardless of external perturbations such as mechanical loading or extreme external stimuli. This is challenging for most current designs since they could be easily snapped back upon applying external loading (*2*–*4*, *21*, *24*, *29*–*31*). Second, previous designs are hard to (re)program at the single bit level, i.e., an individual bistable unit, because the deformation in the unit could be coupled with its neighboring units due to the deformation compatibility (*17*, *23*). Recent study on tileable mechanical computing metamaterials shows the promising reprogrammability with stable memory at the unit-cell level (*23*). It uses magnetic actuation to reversibly and independently switch each binary element made of a bistable shell (*23*). Third, most studies are limited to binary information processing (*2*–*4*, *7*, *20*, *21*, *23*, *26*, *28*–*30*). How to increase the information densities beyond binary information by reprogramming binary states to more states remains challenging and unsolved.

**Fig. 1.**
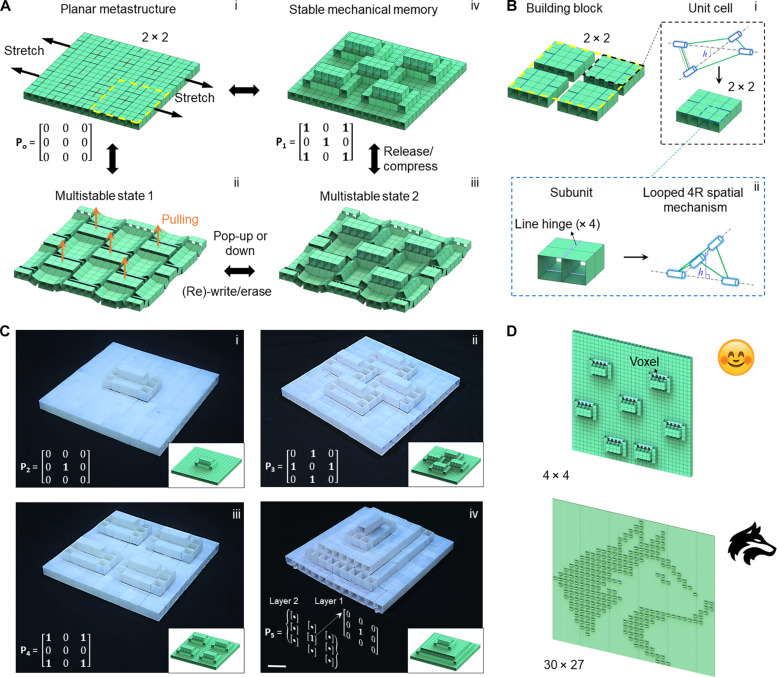
Multistable and transformable metastructures with stable and high-density mechanical memory. (**A** and **B**) Schematics of binary information (re)-writing and stably storing in a planar hierarchical metastructure composed of 2 × 2 building blocks [(A), i]. Each block consists of 2 × 2 unit cells and four-cube subunits (B) connected in hierarchical closed-loop mechanisms. Uniaxially stretching the metastructure leads to a multistable corrugated structure [(A), ii]. It is composed of 3 × 3 bistable elements with each element capable of independently popping up (denoted as 1) or down (denoted as 0) under out-of-plane mechanical or magnetic actuations. Each element acts as an independent binary bit for information writing and erasing under the stretched state (ii and iii). Strain-releasing and slight compression lead to a temporarily locked and compact configuration that stores the information (3 × 3 matrix P_i_) stably regardless of external mechanical or magnetic perturbations (iv). Re-stretching makes the stored information become editable for rewriting or formatting. (**C**) Schematics and the representative optical images of reprogrammed multistate configurations for combinatorial information storage from the platform in (A). (i) to (iii) show the selective pop-up or down and (iv) shows the transformation into a multistory configuration capable of storing multidimensional information. Scale bar, 4 cm. (**D**) Schematics of illustrated application of the bistable element as a pop-up voxel for information display in two examples of a smiley face (top, 4 × 4 platform) and a wolf head (bottom, 30 × 27 platform).

Here, we report mechanism-based multistable multifunctional mechanical computing metastructure with stable, high-density mechanical memory and high reprogrammability. The metastructure is constructed from hierarchical planar tessellation of reconfigurable rigid cube–based building blocks [Fig. 1A (i)]. Each building block is composed of 2 × 2 unit cells and subunits (Fig. 1B). Cubes are bonded at their edges through elastic rotational hinges to form flexible closed-loop mechanisms in both building blocks and unit cells for shape reconfiguration (Fig. 1B). Uniaxially stretching the planar metastructure leads to both a bifurcated and multistable state with periodic corrugated surface features [Fig. 1A (ii)]. We find that all the ridged segments can be designed to be bistable under the pre-stretched state. Each bistable element can act as an independent binary unit by easily and reversibly popping up (“1” state) or down (“0” state) via snapping under out-of-plane mechanical or magnetic actuations [Fig. 1A (iii) and fig. S1, A to C]. Such physical binary elements can be used for combinatorial information writing [Fig. 1, A (iv) and C (i to iii)], erasing, reading, and encryption, as well as voxels for information display (Fig. 1D). The information can be stably stored by releasing the pre-strain [Fig. 1A (iv) and fig. S1D]. Beyond binary units, the bifurcated metastructure can be further reprogrammed into multilevel stepwise pop-up structures for storing multidimensional information [Fig. 1C (iv)].

To better understand the mechanical memory, we first investigate the reconfiguration kinematics of both the composed unit cells and building blocks, as well as the bistable behavior in the building block through both modeling and experimental studies. Then, we explore the remote magnetic actuation of the periodic tessellated metastructure at the single-bit level for binary and beyond-binary information processing and storage. Last, we explore the applications of the metastructure platform in information encryption and mechanical computing as logic gates.

## RESULTS

### Shape transformation in the unit cell

**Fig. 2.**
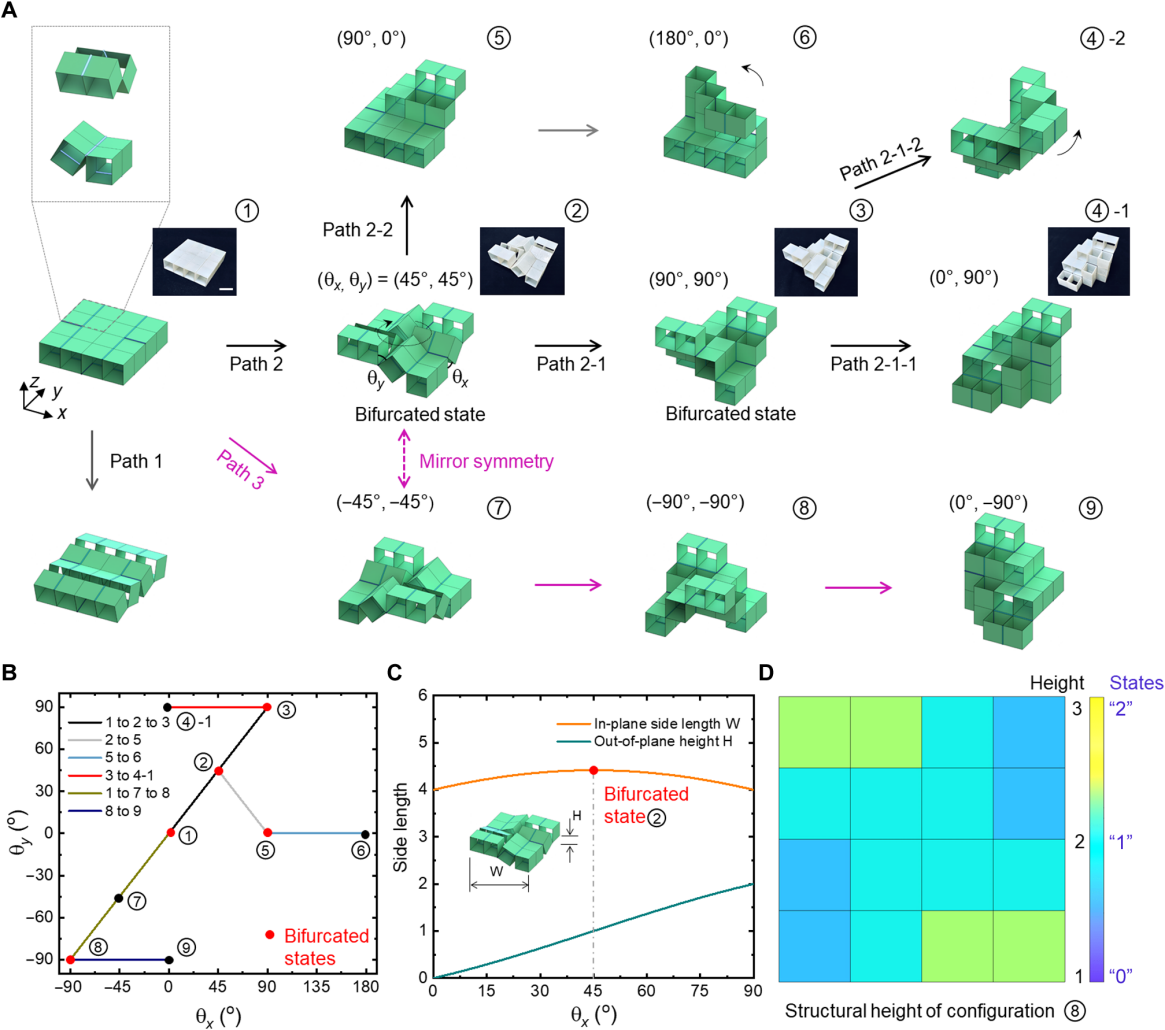
Versatile transformable unit cell with high-density information storage. (**A**) The unit cell follows multiple paths to transform into over 10 distinct architected configurations. Insets show the representative prototypes. Path 1 follows simple chain-like shape changes. Path 2 and path 3 are mirrored about *xy* plane and follow the bifurcated paths to transform into multistory architectures. The shape transformation can be characterized by the two rotation angles of θ*_x_* and θ*_y_*. (θ*_x_*, θ*_y_*) marks the rotation angles in each transformed configuration. Scale bar, 2 cm. (**B**) The angle relationships between θ*_x_* and θ*_y_* during the shape transformation in (A). (**C**) The changes in the dimensions of width *W* and height *H* with θ*_x_* during the transformation from configuration ① to bifurcated configurations ② and beyond. The peak point in W marks the bifurcated state. (**D**) An in-plane mapping height contour by uniquely projecting the height of each cube in the transformed multistory architectures in ⑧ to a 2D 4 × 4 array. The contour shows the height of each cube and the respective three states of 0, 1, and 2 as ternary bits for information processing.

As shown in Figs. 1B and 2A, the hierarchical building block is composed of 2 × 2 unit cells [Fig. 1B (i and ii); see more details in figs. S2 and S3]. Each unit cell consists of 2 × 2 subunits. Each subunit is constructed by symmetrically connecting four rigid cubes with four elastic line hinges into an overconstrained system. The prototypes were made by bonding the 3D printed rigid polymer cubes with ultra-adhesive plastic tapes (see Materials and Methods for details). The subunit can be treated as a 4R (4: number of rigid links, i.e., cubes; R: rotatable joints, i.e., hinges) rigid closed-loop kinematic mechanism [Fig. 1B (i and ii)]. Similarly, the unit cell can be considered as a 4R closed-looped mechanism but flexible, which is composed of four flexible links (i.e., four subunits) connected by four symmetrical line hinges, generating a twofold structural symmetry [Fig. 1B (i)]. The subunit can only deform as a chain-like configuration either along *x* or *y* axis [Fig. 2A (i) and movie S1]. However, the unit cell can undergo deformation with both in-plane expansion and out-of-plane extrusion (movie S1), forming an internal structural loop in the center (marked by the black-colored circular arrow in Fig. 2A, ②).

The unit cell exhibits rich shape transformations with over 10 multistory configurations by following three different paths as shown in Fig. 2A, i.e., path 1 simply reconfiguring as a chain-like mechanism [Fig. 2A (i)], and the other two similar path 2 and path 3 as a looped rigid mechanism [Fig. 2A (ii) and movie S1]. Because of the structural symmetry, the reconfigured shapes in path 2 and path 3 show a mirror symmetry about *xy* plane (Fig. 2A). All the shape changes can be characterized by the two rotation angles (θ*_x_*, θ*_y_*) at the boundary hinges along *x* and *y* axis, respectively (see details in Fig. 2A, ②). Figure 2B shows the corresponding angle relationship, i.e., kinematics, during shape changes in Fig. 2A, where all the shape transformation follows simple linear relationship. Specially, Fig. 2B shows two branched points at (θ*_x_*, θ*_y_*) = (45°, 45°) (i.e., configuration ② in Fig. 2A) and (θ*_x_*, θ*_y_*) = (90°, 90°) (i.e., configuration ③ in Fig. 2A), which represent kinematic bifurcation points with their angles predicted by the Denavit-Hartbenberg rule (figs. S4 to S6) (*38*–*40*). We note that during transforming from configuration ① to ③ along the path 2-1, i.e., θ*_x_* increases from 0° to 90°, the nominal in-plane side length *W* (see the inset of Fig. 2C) of the unit cell first increases nonlinearly and reaches its peak at the bifurcated configuration ② (i.e., θ*_x_* = 45°) and then symmetrically decreases to the original length, whereas the out-of-plane structural height *H* keeps increasing monotonically (Fig. 2C). As demonstrated later, this unique in-plane expansion and contraction of the unit cell intrinsically provide the physical basis for achieving bistable deformation in the building block.

Next, we explore leveraging the rich transformed architectures for information processing. The unit cell has 16 cubes. We project the height of each cube in the transformed multistory architectures to a 2D 4 × 4 array to form an in-plane mapping height contour. Specially, we observe that for the bifurcated configuration ③ or its mirrored configuration ⑧, each cube in the unit cell has a unique mapping height varying from 1 (i.e., story 1) to 3 (i.e., story 3), as shown in Fig. 2D. When compared to its original uniform mapping height of 1 before transformation, it gives a height difference varying from 0, 1, and to 2 in each cube, which can be assigned a respective "0," "1," and "2" state for potentially increasing information density beyond binary as discussed later. In contrast, most of the other transformed architectures do not have such unique one-to-one mapping height due to the stacking or overlapping of the cubes (e.g., configuration ④-1, ④-2, and ⑥). Thus, we can simply use the unique in-plane mapping height contour to represent the transformed 3D architecture for information processing.

### Transformable and bifurcated building blocks with high-density information memory

**Fig. 3.**
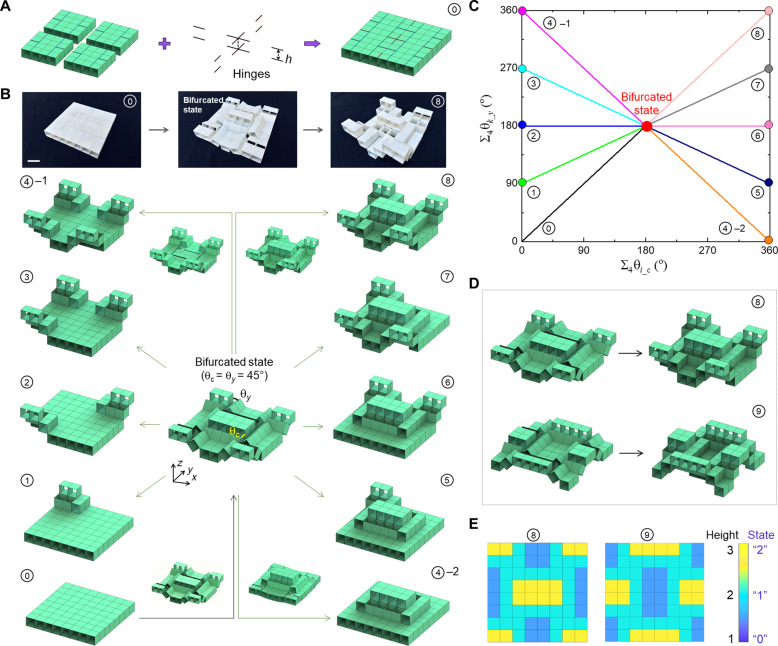
Versatile transformable building block with high-density information storage. (**A**) Schematics of constructing the hierarchical planar building block by connecting four unit cells with 16 line hinges. (**B** and **C**) Versatile postbifurcated transformed configurations from the bifurcated state in the center through combinatorially lifting up the extruded segments around the boundary or center or combined. The related transformation kinematics on the change of the rotation angles are shown in (C). The top row of (B) shows the representative shape changes in the prototype from configuration ① to configuration ⑧ bypassing the bifurcated configuration. Scale bar, 2 cm. (**D** and **E**) Schematic illustration of two selected mirrored transformations in ⑧ and ⑨ with their unique projected in-plane mapping height contour shown in (E).

As shown in Fig. 3A (i), given the twofold structural symmetry of the unit cell, we create the building block with fourfold structural symmetry by combining four unit cells with 16 line hinges. The hinges are symmetrically placed on the top and bottom surfaces to constrain the building block with a minimum number of structural degrees of freedom (DOF), as well as to preserve the reconfiguration paths in the unit cell. Figure 3B shows the collection of transformable configurations of the building block. Similarly, its transformed shapes can be characterized by the combinatorial rotation angles (θ*_x_*, θ*_y_*) in the four unit cells. (θ*_x_*, θ*_y_*) in the unit cell now becomes (θ_c_, θ*_y_*) with θ_c_ and θ*_y_* defined as the rotation angle at the center and the corner of the building block (see Fig. 3B), respectively. Similar to the path 2 or path 3 in the unit cell, as θ_c_ = θ*_y_* increases from 0° to 45°, the building block transforms into a corrugated configuration with five out-of-plane extruded segments, i.e., 4 in the corners and 1 in the center. At θ_c_ = θ*_y_* = 45° in each unit cell, it transforms into a bifurcation state (Fig. 3B).

Starting from the bifurcation state, we note that all the five extruded segments can deform independently and compatibly by either further “lifting up” or “pushing back” without interference. Thus, the building block exhibits largely enhanced reconfigurability through different combinations of its five independently deformable structural segments (movie S2). To explicitly describe each reconfigured paths, we define the angle-sum pair (∑_4_θ_*i*_c_, ∑_4_θ_*k*_*y*_) with *i* and *k* representing the unit cell number from 1 to 4. At the bifurcated state, we have (∑_4_θ_*i*_c_ = 180° and ∑_4_θ_*k*_*y*_ = 180°). Figure 3B shows nine selected combinatorial transformed shapes using two different reconfiguration ways. One is to selectively further lifting up *m* (*m* = 1, 2, 3 or 4) corner structural parts while pushing back the central part with ∑_4_θ_*i*_c_ = 0°, e.g., configurations ①, ②, ③, and ④-1 on the left column. The other is to further lift up both the central and *m* corner structural parts with ∑_4_θ_*i*_c_ = 360°, e.g., configurations ④-2, ⑤, ⑥, ⑦, and ⑧ on the right column. Figure 3C shows the corresponding transition paths with simple linear angle relationships. For example, configurations ① and ④-2 show only one elevated segment at the corner with (∑_4_θ_*i*_c_ = 90° and ∑_4_θ_*k*_*y*_ = 0°) and in the center with (∑_4_θ_*i*_c_ = 360° and ∑_4_θ_*k*_*y*_ = 0°), respectively, whereas all the central and corner segments are elevated in configuration ⑧ with (∑_4_θ_*i*_c_ = 360° and ∑_4_θ_*k*_*y*_ = 360°).

Similarly, by following path 3 of the unit cell in Fig. 2A, we can get mirrored transformed configurations of Fig. 3B. For example, Fig. 3D show the shape of configuration ⑧ and its mirrored counterpart configuration ⑨ via path 3 (see more details in fig. S7), respectively. We find that all the transformed configurations show the unique one-to-one mapping height contours in the 2D 8 × 8 array; see Fig. 3E for configurations ⑧ and ⑨ for example. Comparing to the unit cell, the building block could have much higher information densities due to its rich reconfigurability. The building block can achieve 30 (2 × 2^4^ − 2) distinct transformed configurations and thus 30 2D mapping patterns for processing and storing information.

### Bistable building block

**Fig. 4.**
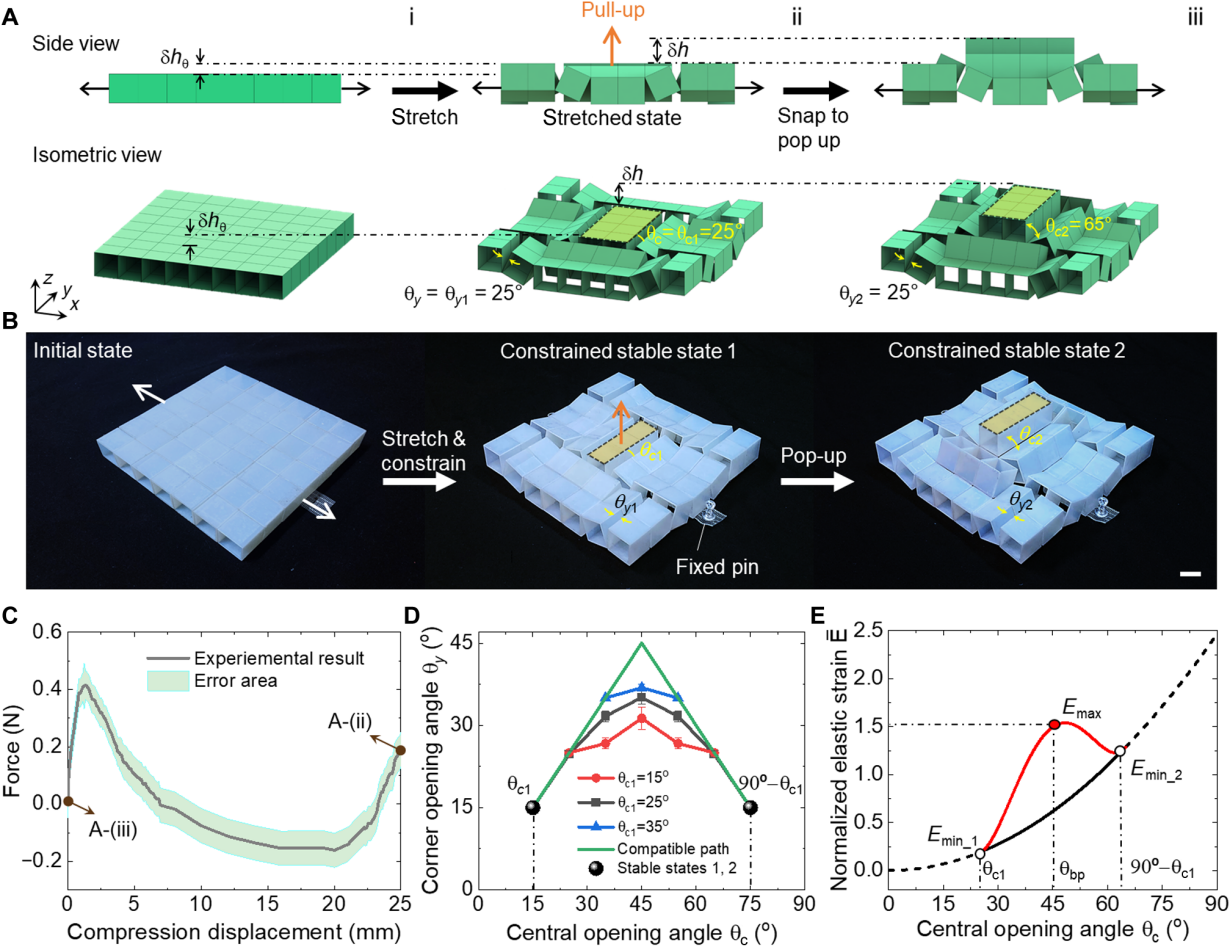
Bistability in the constrained building block (**A**) Schematic illustration of achieving bistability in the building block. Uniaxially stretching the block (i) first to render a constrained stable state 1 under the pre-stretched state (ii). Pulling up the center segment snaps to the other pop-up constrained stable state 2 (iii). (**B**) Experimental demonstration of the bistability in the constrained building block using boundary fixed pins to retain the pre-stretched state. Scale bar, 2 cm. (**C**) Measured compression force-displacement curve in the constrained building block at the stable state 2. (**D**) Comparison of the central opening angle θ_c_ versus corner opening angle θ*_y_* between the constrained and unstrained building blocks with different θ_c1_. (**E**) Comparison of the normalized energy versus θ_c_ between the constrained and unstrained building block with θ_c1_ = 25°.

In addition to the independent combinatorial shape transformation features, the building block can also achieve bistable deformation modes. As illustrated in Fig. 4A (i), uniaxially stretching the building block (e.g., along the *x* axis) generates a corrugated structure. Upon fixing the boundary parts as shown in the prototype of Fig. 4B, the nonbifurcated corrugated configuration with θ_c_ = θ*_y_* < 45° under the pre-stretched state can stably stay. Figure 4A (ii) shows an example of a stable pre-stretched configuration with θ_c1_ = θ_*y*1_ = 25° under pinned constraints. Then, vertically pulling the central part leads to snapping through to the other stable state with a pop-up central segment; see the configuration in Fig. 4A (iii) with an elevated height of δh compared to the configuration in Fig. 4A (ii). θ_c2_ in the stable state 2 increases to 65° with θ_c1_ + θ_c2_ = 90°, while θ_*y*2_ = θ_*y*1_ = 25° remain unchanged. Note that the bifurcated state with θ_bp_ = 45° represents an unstable state as discussed next.

To experimentally examine the bistability, we use a fiber-enhanced ultra-adhesive tape as the line hinges to facilitate the strain energy stored in the boundary hinges under the pre-stretched state (see Materials and Methods for details). Figure 4B shows the bistable demonstration by manually pulling and pushing the central segment under a fixed pre-stretched state by two pins (movie S3). Through the displacement-control uniaxial mechanical test method, we further validate such bistable deformation by vertically compressing the sample from its second stable state back to the first stable state (see fig. S8). The measured force-displacement curve in Fig. 4C shows a sudden force drop that corresponds to the occurrence of snap-through instability, followed by a large negative force area, indicating the bistability.

We combine experiments and simplified modeling to understand the underlying mechanism of the observed bistability. Considering the expansion-contraction reconfiguration in the unit cell with free boundary in Fig. 2C, we hypothesize that the bistability relies on the incompatible reconfiguration kinematics between the central θ_c_ and corner opening angle θ*_y_* of the unit cells, as well as the coupled modified kinematics and elasticity induced by the fixed boundaries. To validate it, we measure the variation of θ*_y_* as a function of θ_c_ under fixed boundaries and compare it to the boundary-free compatible path. Figure 4D shows that for all the initial confined stable configurations with pre-rotational angle θ_c1_ = 15°, 25°, and 35° under different pre-stretched strains, θ*_y_* under boundary constraints is always smaller than that of the boundary-free compatible path, showing the distinct kinematics under constrained conditions. Consequently, during the bistable switch, it needs to overcome the energy barrier from the elasticity in the building blocks that is absent in rigid mechanisms shown in Fig. 4E, arising from the incompatible out-of-plane deformation between the central and boundary structural parts discussed next.

### Coupled kinematics-mechanics modeling of the bistable building block

**Fig. 5.**
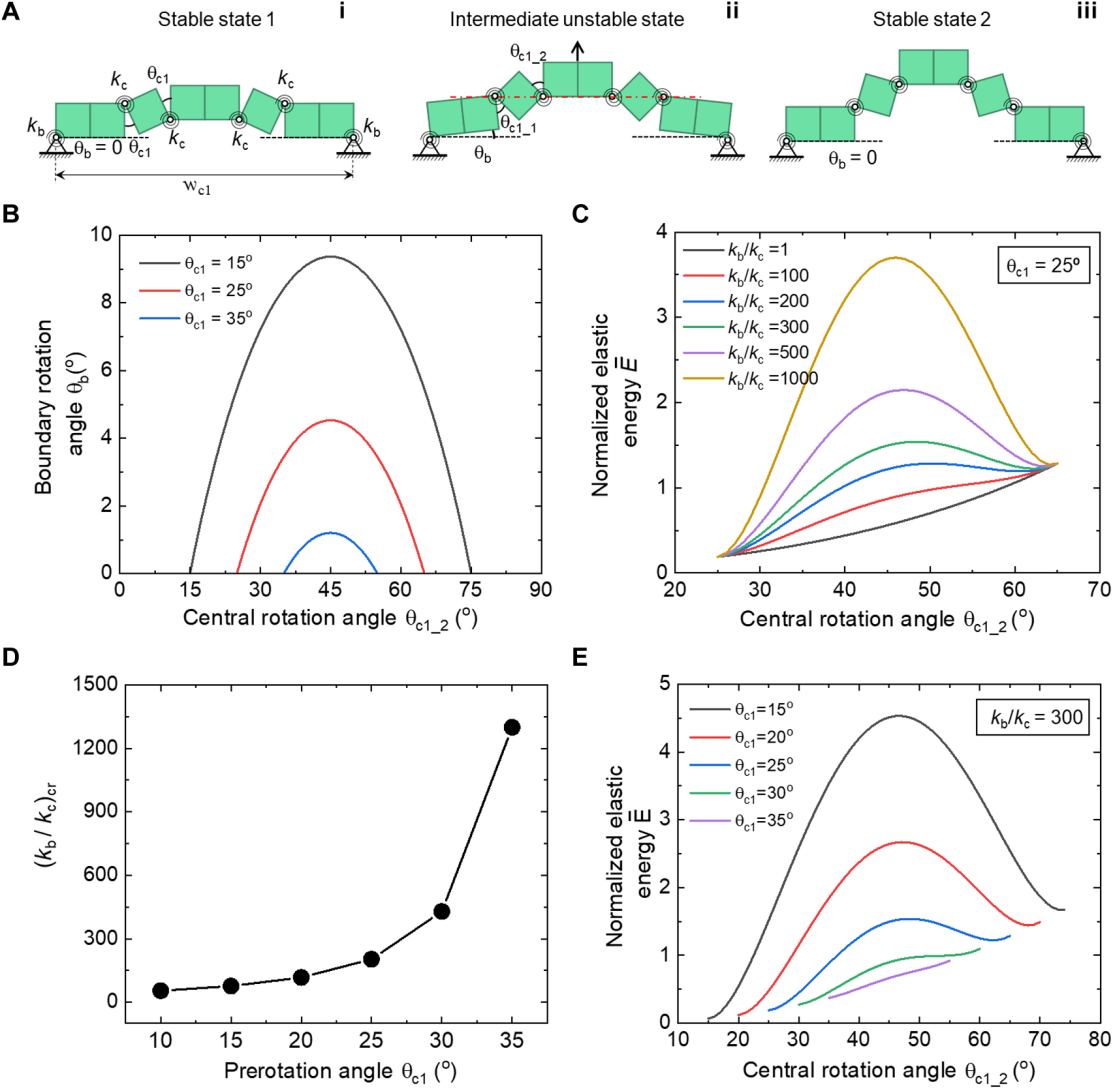
Theoretical analysis for the structural bistability of the constrained building block (**A**) Schematic illustration of the simplified 1D kinematics-mechanics model of the constrained building block with elastically torsional central (*k*_c_) and boundary (*k*_b_) hinges. (**B**) The variation of the boundary rotation angle θ_b_ as a function of the center rotation angle θ_c1_2_ and the pre-rotation angle θ_c1_. (**C**) Energy landscape (the normalized elastic energy) as a function of torsional stiffness ratio *k*_b_/*k*_c_ at θ_c1_ = 25°. (**D**) The critical values of stiffness ratio (*k*_b_/*k*_c_)_cr_ for enabling bistability with different θ_c1_. (**E**) Energy landscape as a function of θ_c1_ at *k*_b_/*k*_c_ = 300.

To uncover the effects of the boundary constraints and the pre-stretched strain on the kinematics and bistability, we further develop a simplified 1D coupled kinematics-mechanics model based on the stored elastic energy shown in Fig. 5A and fig. S9. Each cube is assumed to be rigid and connected by elastic torsional springs with the same torsional stiffness *k*_c_. The torsional stiffness of the boundary hinges is *k*_b_. Thus, all the elastic energies are assumed to be stored in the elastic hinges.

For the general case of an intermediate state in Fig. 5A (ii), its elastic strain energy *E* normalized by *k*_c_, i.e., $\bar{E}$ , can be expressed as

$$\bar{E}=E/k_{c}=\left(k_{b}/k_{c}\right)\theta_{b}^{2}/2+\theta_{c1\_1}^{2}/2+\theta_{c1\_2}^{2}/2$$ (1)

where θ_b_ is the rotation angle of the boundary cube. θ_c1_1_ and θ_c1_2_ are the left and right rotational angles of the central cube, respectively. These three angles are dependent on each other since the bistable switch is a single DOF motion due to the boundary constraints, i.e., the fixed distance *w*_c1_ between the two pinned points. From simple geometry in Fig. 5A (ii), we have.

$$\theta_{b}+\theta_{c1\_1}=\theta_{c1\_2}$$ (2)

$$w_{\text{c1}}=2l\;\left[1+\sqrt{5}\text{cos}\left(\theta_{0}+\theta_{b}\right)+{\text{cos}\theta }_{c1\_2}+{\text{sin}\theta }_{c1\_2}\right]$$ (3)

where *l* is the cube side length and θ_0_ = cos^−1^($\sqrt{5}$ ). For both stable states shown in Fig. 5A (i and iii), we have θ_b_ = 0 and thus θ_c1_1_ = θ_c1_2_. Specially, at the initial stable state 1, we have θ_c1_1_ = θ_c1_2_ = θ_c1_, i.e., the prerotational angle, and Eq. (3) reduces to

$$w_{\text{c1}}=2\;l\;\left(3+{\text{cos}\theta }_{\text{c1}}+{\text{sin}\theta }_{\text{c1}}\right)$$ (4)

The nominal pre-strain ε_pre_ is defined as ε_pre_ = (*w*_c1_ − *w*)/*w* with *w* = 8 *l*, which gives

$$\varepsilon_{\text{pre}}=\left({\text{cos}\theta }_{\text{c1}}+{\text{sin}\theta }_{\text{c1}}-1\right)/4$$ (5)

Since we have the same fixed distances in Eqs. 3 and 4, thus, for a given pre-rotational angle θ_c1_ or equivalently ε_pre_, the single DOF motion can be described by the central rotational angle θ_c1_2_ by combining Eqs. 2 to 4, i.e.,

$$\theta_{b}={\text{cos}}^{-1}\;\left(2+{\text{cos}\theta }_{\text{c1}}+{\text{sin}\theta }_{\text{c1}}-{\text{cos}\theta }_{c1\_2}-{\text{sin}\theta }_{c1\_2}\right)/\sqrt{5}-\theta_{0}$$ (6)

Figure 5B shows the variation of θ_b_ as a function of θ_c1_2_ under different θ_c1_ (or equivalently ε_pre_). As θ_c1_2_ increases, θ_b_ shows a peak value (θ_b_)_max_ at the unstable state with the identical value of θ_c1_2_ = 45° for all the pre-rotational angles. As θ_c1_ increases from 15° to 35° (equivalently, ε_pre_ increases from 5.6 to 9.8%), (θ_b_)_max_ markedly decreases from 9.4° to 1.2°.

On the basis of the uncovered constrained kinematics, the normalized strain energy $\bar{E}$ at an intermediate state can be readily obtained by substituting Eqs. 2 and 6 into Eq. 1. $\bar{E}$ is independent of the cube size *l* and is a function of the stiffness ratio *k*_b_/*k*_c_, the pre-rotational angle θ_c1_ or ε_pre_, and the central rotation angle θ_c1_2_, i.e., $\bar{E}$ = $\bar{E}$(*k*_b_/*k*_c_, θ_c1_, θ_c1_2_). We first compare $\bar{E}$ under both compatible and incompatible kinematic paths in Fig. 4E for the building block with θ_c1_ = 25° (ε_pre_ = 8.2%) and *k*_b_/*k*_c_ = 300. For the compatible kinematic path under free boundary, $\bar{E}$ increases monotonically with θ_c_ = θ_c1_2_. In contrast, for the incompatible kinematic path under constrained boundary, $\bar{E}$ shows a much higher strain energy and two local minimums (*E*_min_1_ and *E*_min_2_), which correspond to the initial and snapped stable states with *E*_min_2_ > *E*_min_1_ > 0, respectively. The peak point corresponds to the unstable state, which is located at the bifurcation state with an angle of 45° indicating the unstable structural characteristic. Energetically, the bistability is attributed to the competition of the stored elastic energy between the boundary *E*_b_ and central hinges *E*_c_ during the bistable switch under constrained boundaries as seen from Eq. 1, where *E*_b_ first increases and then decreases with θ_c_, whereas *E*_c_ increases monotonically with θ_c_.

Next, we explore the effects of the stiffness ratio and pre-strain on the energy landscape of the bistable building block. Figure 5C shows the variation of $\bar{E}$ as a function of *k*_b_/*k*_c_ at a given pre-strain ε_pre_ = 8.2% with θ_c1_ = 25°. As *k*_b_/*k*_c_ increases from 1 to 1000, the energy landscape transits from stable to monostable, and to bistable states with markedly increasing energy barriers. At *k*_b_/*k*_c_ = 1, $\bar{E}$ increases monotonically with θ_c1_2_ and shows a monotonically increasing positive slope, indicating a stable state. As *k*_b_/*k*_c_ further increases to 100, $\bar{E}$ increases monotonically with θ_c1_2_ but shows a local minimum in the slope, indicating a monostable state. As *k*_b_/*k*_c_ further increases to 200 and above, $\bar{E}$ exhibits one peak with zero slope and two local minimums, indicating a bistable state. Thus, for a given pre-strain, a higher stiffness ratio *k*_b_/*k*_c_ will enhance the bistability with a higher energy barrier, whereas a smaller stiffness ratio will facilitate monostability. This is qualitatively validated by the experiment shown in fig. S8F. It shows that increasing the number of the layered tapes that act as elastic hinges to bond the central cubes, i.e., increasing *k*_c_, leads to the transition from a bistable to monostable state. This is consistent with the theoretical prediction since increasing *k*_c_ results in a decreasing *k*_b_/*k*_c_. When the building blocks are tessellated periodically in both directions, the surrounding interconnected cubes will impose stronger boundary constraints since the expansion and out-of-plane rotation of the building block are constrained during the bistable switch. Consequently, tessellation will enhance the bistability and require a higher buckling force to trigger the bistable switch as validated and discussed next.

Figure 5D shows the critical value of *k*_b_/*k*_c_ for bistability, i.e., (*k*_b_/*k*_c_)_cr_, under different pre-strain or pre-rotational angle θ_c1_. It shows a *J*-like curve. As θ_c1_ increases from 10° to 35°, (*k*_b_/*k*_c_)_cr_ increases highly nonlinearly and markedly from 54 to 1300, indicating much stronger boundary constraints required to enable bistability for increasing pre-strains. Figure 5E shows that for a given stiffness ratio, e.g., *k*_b_/*k*_c_ = 300, similar transitions from bistable to monostable and to stable states can be obtained by increasing the pre-rotational angle θ_c1_ or the pre-strain ε_pre_ with markedly reduced energy barrier. Thus, for a given stiffness ratio, a smaller pre-rotational angle or pre-strain will enhance the bistability. This is consistent with the experimental observation shown in fig. S8E. It shows that as θ_c1_ decreases from 35° to 15°, the critical buckling force in the force-displacement curves increases, indicating higher energy barrier and stronger bistability.

### Reprogrammable mechanical metastructure with stable mechanical memory

**Fig. 6.**
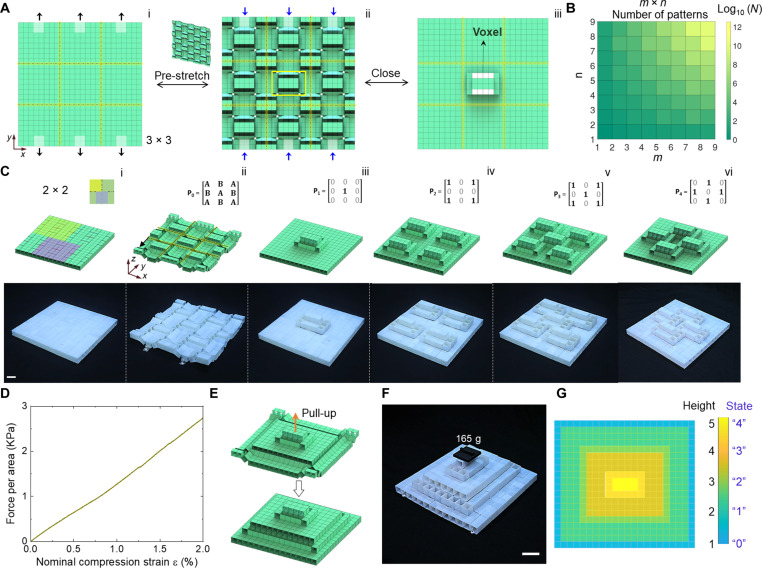
Reprogrammable multistable metastructure with stable and high-density mechanical memory. (**A**) Schematic illustration of a multistable metastructure composed of 3 × 3 building blocks by following stretching, release, and compression to selectively edit and store binary bits information. The popped element can also act as a voxel for display. (**B**) Illustration of a large number of *N* = 3 × 2^2*mn*^ − 1 combinatorial binary states for information (re)writing and (re)erasing in a *m* × *n* metastructure with high information densities. (**C**) Schematic and experimental demonstrations of four selected combinatorial binary states in a 2 × 2 metastructure. Scale bar, 2 cm. (**D**) Uniaxial compression test on the configuration in (C) (iii) to show the compression stability and resistance. A linear compression pressure versus strain is shown. (**E**) Schematic illustration of generating a multistory architectured configuration by pulling the central part. (**F**) Experimental demonstration of the stable multistory configuration under a certain load. Scale bar, 4 cm. (**G**) The unique projected in-plane mapping height contour of the multistory configuration in (F).

Periodically assembling *m* × *n* building blocks creates a reprogrammable multistable mechanical metastructure (see the representative 3 × 3 design in Fig. 6A). When the chain-like path 1 along both *x* and *y* axis are constrained by bonding the cubes (see the bonded cubes in the zoom-in part of Fig. 6A), the undeformed metastructure has only one DOF and deforms by following path 2 (or equivalently path 3) of the unit cell shown in Fig. 2A (ii). Given the kinematic bifurcation and bistable deformation in each building block, we find that all the local out-of-plane extruded structural elements in a uniaxially pre-stretched metastructure are bistable, which can reversibly and independently pop up ("1" state) or pop down ("0" state) via snapping. Thus, an *m* × *n* metastructure can generate a number of 3 × 2^2*mn*^ − 1 different combinatorial binary states for information (re)writing and (re)erasing with high information densities (Fig. 6B).

Figure 6C shows four selected combinatorial binary states in a 2 × 2 metastructure prototype (movie S4). In the prototype, we merged the repeating redundant structural parts among the units to save materials but without affecting their transformation behaviors of the units and the periodic metastructure. The pre-stretched prototype is partitioned into 3 × 3 zones with its binary states represented by a 3 × 3 matrix **P** with **P** = $\left[\begin{array}{ccc} A & B & A\\ B & A & B\\ A & B & A \end{array}\right]$ . A, B = 0 or 1 represents the binary state of flat or pop-up configuration in the segment, respectively. To write the information of **P**_**1**_ = $\left[\begin{array}{ccc} 0 & 0 & 0\\ 0 & 1 & 0\\ 0 & 0 & 0 \end{array}\right]$ into the metastructure, we follow two steps: first is to pull the center segment to snap and pop up (1 state) under the pre-stretched state fixed by the constrained boundaries [see the process from (i) to (ii) in Fig. 6A]; second is to release the pre-stretch to spontaneously flatten the corrugated units (0 state) alongside lateral compression if needed without affecting the pop-up segment, forming a compact configuration [see the process from (ii) to (iii) in Fig. 6A]. Similarly, we can write more binary information in a matrix form into the system with different combinatorial pop-up/down. Furthermore, for the same number of pop-ups, e.g., **P**_**2**_ = $\left[\begin{array}{ccc} 1 & 0 & 1\\ 0 & 0 & 0\\ 1 & 0 & 1 \end{array}\right]$ with four evenly distributed pop-ups throughout the platform, we can also follow path 3 to write different information of **P** = $\left[\begin{array}{ccc} 0 & 1 & 0\\ 1 & 0 & 1\\ 0 & 1 & 0 \end{array}\right]$ with four centered pop-ups shown in Fig. 6C.

We find that the deformed structure with compact configuration is mechanically stable for storing information. We validate this by conducting a compression test based on the pop-up configuration shown in Fig. 6C (iii) with a single pixel (or equivalently information bit). Figure 6D shows its compression force-displacement behavior without boundary constraints. It shows that it can bear about 2.8 kPa pressure at an applied compression strain of 2% without collapse.

Furthermore, given the reversible bistability, we can either selectively or entirely erase the stored information in certain zones of the platform. For example, for selective erasure, we can re-stretch the platform slightly to return to a multistable state, where the platform becomes editable under the re-stretched state. Then, selectively pushing down the pop-ups erases the related stored information. Re-compressing the edited structure returns to the stable state to stably store the edited information without additional energy consumption [e.g., the reverse process from (iii) to (ii) in Fig. 6A and see more details in movies S3 and S4]. Similar to disk formatting, upon applying a relatively larger re-stretching strain close to the bifurcation strain of 10.4%, all the stored information can be erased and the whole structural platform becomes flattened.

The information density can be enhanced by further reprogramming the same metastructure to a multilayer configuration. As shown in Fig. 6E, starting from the configuration with one pop-up in the center, we can continuously pull up the top segment to generate a five-story pyramid-like configuration with stepwise features (see Fig. 6F for the prototype) through a two-step process (see more details in the Supplementary Materials). The five-story metastructure also shows a unique one-to-one mapping height contour in a 2D layout, where each cube represents a single state of either 0, 1, 2, 3, or 4 depending on its height for quaternary information processing (Fig. 6G). The physical prototype stands stable and can tolerate certain external load for stable storage of mechanical memory. As the number of building blocks further increases, the metastructure can achieve much higher information storage density than previous works (*23*, *24*, *30*, *31*) through both the combinatorial independent pop-ups and the higher multistory pyramid-like structures (figs. S10 and S11).

### Information writing under magnetic actuation

On the basis of the reprogrammable mechanical metastructure platform, in the following, we further explore its versatile applications in information storage, information encryption, and mechanical logic computation.

**Fig. 7.**
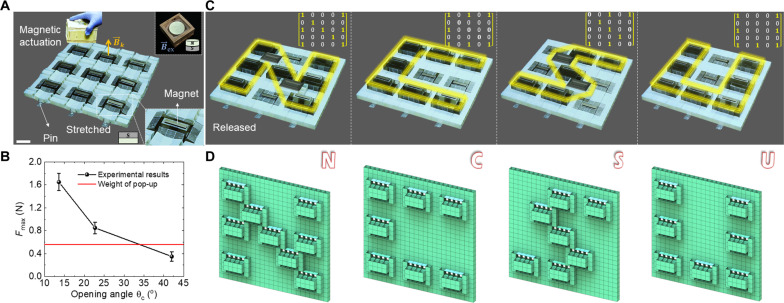
Application of the multistable metastructure for information writing and display. (**A**) Illustration on individual magnetic actuation of binary bits (bistable elements) for writing and erasing in the constrained 3 × 3 metastructure. Nine elements are bonded with magnetic strips on their top surface for remote actuation. Scale bar, 4 cm. (**B**) The maximum pulling/pushing force versus the opening angle θ_c_. Red line shows the self-weight of the pop-up segment. (**C** and **D**) Experimental and schematic demonstrations of mechanical memory editing (four letters N, C, S, and U) or display through remote magnetic field and stable information storage by releasing and compression. The insets show the stored binary information in a 5 × 5 matrix form.

Figure 7A shows the multistep process of generating a single central pop-up strut as a voxel in a 3 × 3 metastructure by following the procedures of pre-stretching, selectively pop-up, and release and compression. The 3 × 3 metastructure renders a 5 × 5 matrix. We note that for different pre-stretched strains ε*_pre_*, unlike the free expansion without constraints, the side length of the constrained metastructure almost remains unchanged during bistable switches of the local structural segments (fig. S12 and movie S4). Moreover, Fig. 7B shows that for the fabricated 3 × 3 prototypes, the maximum pulling/pushing force *F*_max_ to overcome the energy barrier and trigger the snapping in the local structural elements remains low, where *F*_max_ ≈ 1.65, 0.85, and 0.35 N for θ_c_ (ε*_pre_*) = 12.5° (4%), 22.5° (7.2%), and 42.5° (10%), respectively. For the case of θ_c_ = 42.5° (below the theoretical bifurcation angle of 45°), *F*_max_ ≈ 0.35 N is even smaller than its self-weight force (~0.55 N), which means, practically, the pop-up strut cannot stably maintain its second stable state under self-weight. When considering the effect of unavoidable self-weight, we find that the critical bifurcated angle enabling bistability in the prototype is ~33°, which is lower than the theoretical value of 45°.

Given the small *F*_max_ in triggering the independent bistability in the prototype, we further explore using the simple remote magnetic field to actuate the reversible bistable switch (see movie S5) in the metastructure as a mechanical memory storage device for information storage and display (e.g., letters or images). Figure 7 (C and D) shows the schematic and experimental demonstration of writing four letters of “NCSU” onto the 3 × 3 platform through the untethered actuation by using permanent magnets (see more details in the Supplementary Materials). Thin plate–shaped permanents are attached to the top surfaces of nine black-colored local struts [see the right-bottom inset in Fig. 7A (i)]. Under a remote magnetic field (fig. S14), the local struts can be independently and remotely pulled up or pushed down to stay in their stable positions without affecting their neighboring elements, where local supports are not needed for the programming (see figs. S14 and S15 and more details in the Supplementary Materials). The insets of Fig. 7C show the storage of binary information in a 5 × 5 binary matrix for each character (e.g., “N,” “C,” “S,” and “U”), e.g., N = $\left[\begin{array}{ccccc} 1 & 0 & 0 & 0 & 1\\ 0 & 1 & 0 & 0 & 0\\ 1 & 0 & 1 & 0 & 1\\ 0 & 0 & 0 & 1 & 0\\ 1 & 0 & 0 & 0 & 1 \end{array}\right]$ and S = $\left[\begin{array}{ccccc} 0 & 0 & 1 & 0 & 1\\ 0 & 1 & 0 & 0 & 0\\ 0 & 0 & 1 & 0 & 0\\ 0 & 0 & 0 & 1 & 0\\ 1 & 0 & 1 & 0 & 0 \end{array}\right]$  Similarly, more complex patterns or even images can be stored and displayed using the bistable voxels in a large size metastructure platform. For example, a schematic smiley face on a 4 × 4 platform (Fig. 1D, top) and a wolf-head on a 30 × 27 platform (Fig. 1D, bottom). Given the deformation independence of each local structural element, the same metastructure could act as a pluripotent mechanical platform for writing/re-writing and erasing for stable information storage (see movies S6 and S7).

### Information encryption and decryption

**Fig. 8.**
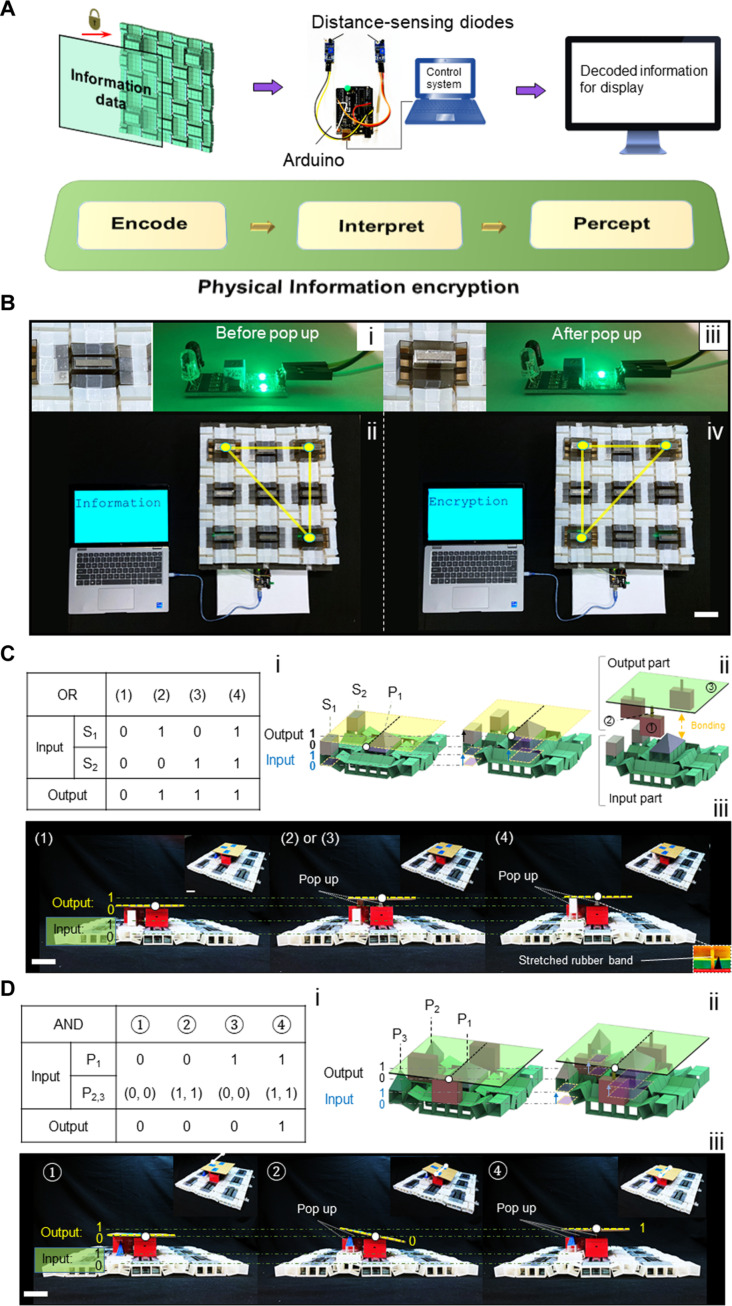
Applications of the multistable metastructure for information encryption and mechanological computing. (**A** and **B**) Schematic illustration and experimental demonstration for information encryption and decryption via distance sensing and display by encoding the word information into the unique pop-up geometrical shapes in the bistable elements, e.g., a right diagonal triangle represents the word of “Information” (ii). Scale bar, 4 cm. (**C** and **D**) Schematic illustration and experimental demonstration for logic gates of OR (C) and AND (D). Scale bar, 4 cm. Demonstration of other binary logic computation of NOR and NAND is shown in fig. S19.

Next, we further exploit the metastructure as a structure-based information encryption (SIE) device by purposefully encoding distinct information onto this multistable system (see more details in Materials and Methods). Figure 8A shows the working mechanism for the designed SIE system. First, considering each pop-up strut as one single vertex, we can encode the encrypted information onto the uniquely patterned pop-up struts in the form of vertices, lines, and/or polygons. Second, we can decipher these patterns with an external reading system (e.g., a screen display) for information decryption and display (see more details in fig. S16A). Figure 8B shows one example of encrypting the word “Information” into a uniquely pop-up right-angle triangle in the metastructure [Fig. 8B (ii) and fig. S16B]. The three pop-up struts represent the three vertices of the triangle, which corresponds to $\left[\begin{array}{ccccc} 1 & 0 & 0 & 0 & 1\\ 0 & 0 & 0 & 0 & 0\\ 0 & 0 & 0 & 0 & 0\\ 0 & 0 & 0 & 0 & 0\\ 0 & 0 & 0 & 0 & 1 \end{array}\right]$ in the binary matrix. An electronic interpreting system with displacement-sensing diodes and Arduino circuit board [Fig. 8B (i and iii), and see more details in fig. S16A, right] will detect and transform these physical information into unique electric signals. These electric signals can be captured and deciphered by the control system to display on a computer screen. When the patterning of the pop-up struts does not match the encoded one, it will show “Encryption,” e.g., the left pop-up triangle shown in Fig. 8B (iv) (movie S8).

Given the distinct spatial locations of each structural element and their independent combinations, we note a large volume of information can be encoded into our proposed metastructure platform even with a small number of units. For example, on a simple 3 × 3 platform, the potential information capacity can be calculated as *N*_info_ = 9! − 1 = 362,879 (for a *n* × *n* platform, *N*_info_ = *n*^2^! − 1), allowing encoded high-capacity information in small areas. Thus, compared to previous designs (*23*, *24*), our proposed system shows superior advantages in terms of simple structural forms, easy fabrication and actuation, and superior informational encryption capability.

### Mechanical logic gates

Last, we explore the metastructure as simple mechanical logic gates. Figure 8 (C and D) demonstrates the achievement of both “OR” and “AND” logic gate operations by using independent bistability in local elements. To facilitate the reading of output information (see more details in fig. S17A and the Supplementary Materials), we use a supported height-adjustable flat plate on the top to cover a small region of the platform. Its initial state is set as an output of “0.” When the plate is even and elevated, it outputs "1," otherwise "0" for the cases of either being tilted or lowered. The configurations of the top plate are determined by the pop-up ("1") or pop-down ("0") motions of three supports bonded to the bistable elements as inputs. A pyramid support denoted as P_1_ is placed in the center with two other neighboring supports surrounded, e.g., cuboids of S_1_ and S_2_ and pyramids of P_2_ and P_3_ for the OR and AND logic gate, respectively.

Figure 8C and fig. S17B show that when P_1_ is popped up and fixed, popping-up either S_1_ or S_2_ or combined as inputs leads to a stable and evenly elevated plate on the top as an output of "1" for an OR operation, because one point contact at P_1_ alongside one plane contact at S_1_ or S_2_ will render a stable and even surface. For the case of AND logic gate shown in Fig. 8D and fig. S17C, three pyramid that supports P_i_ are free to pop up or down, providing the point contacts to support the top plate. Only when the plate is supported by three pop-up point contacts, i.e., P_1_ = P_2_ = P_3_ = 1, it will generate a stable and evenly elevated plate as an output of "1" for an AND operation.

We note that most previous mechanical logic metastructures are limited to 1D and 2D structural forms (*1*–*3*, *11*, *19*, *20*, *26*, *29*, *41*). Our design extends the structural form of the mechanical binary logic computation to a 3D structural form. In Fig. 8 (D and E), we demonstrate the logic operation in only one zone. In particular, given the independent bistability of each local elements, such design principles can be readily applied to multiple zones for conducting a myriad of parallel mechanical binary operations on the same metastructure platform (see details in fig. S18). Moreover, by altering the structural components as schematically illustrated in fig. S19, we can also conduct “NOR” and “NAND” binary logic computations in our designed platform.

## DISCUSSION

In summary, we proposed a reprogrammable multistable mechanism-based metastructure composed of planar and compact tessellation of reconfigurable cube-based building blocks. We explored its potential as a pluripotent reconfigurable mechanical computing platform through both experimental testing and proof-of-concept demonstrations. In sharp contrast to the pluripotent reconfigurable kirigami sheet based on the kinematics of rigid folding (*42*), our platform relies on the coupling between the kinematics and elasticity for reconfigurability and multistability. Kinematic bifurcation in the building blocks enables combinatorial structural reconfigurability and bistability of the local elements for enhanced deformation re-programmability, where the bifurcated state corresponds to an unstable state. The bistability in the local elements can be manipulated independently without interference with their neighboring elements for information storage and computing at the single bit level under both contact-based mechanical forces and remote magnetic actuation. Leveraging both kinematic bifurcation and bistability characteristics, we demonstrated the potential applications in selectively information writing, erasing, and high-density stable memory storage, as well as information encryption and logic gates.

Given the scale independence of the coupled kinematics-mechanics designs, we envision that the uncovered design principles can be applied to both rigid and soft building blocks with small- or large-scale sizes. We observed similar bistability in the soft building blocks either by replacing the rigid cubes with paper-based cubes that can be easily sheared (fig. S20) or using planar thick soft elastomeric plates (fig. S21). Stretching the planar thick rubber plate with segmented blocks and hinges via laser cutting leads to popping up into a similar 3D multilayer structure, which exhibits similar bistability under a pre-stretched state (fig. S21 and movie S9). This is similar to stretching-induced pop-up of bistable structs in thin kirigami sheets (*43*, *44*) but with distinct architectured blocks in our segmented thick plates. Such designs facilitate fabrication at small scales without the need for cube assembly, which could find potential applications in small-scale mechanical memory devices with robust and high stored information densities, as well as other applications including micro-electromechanical system, haptic devices, and reconfigurable metasurfaces for acoustic wave guide, particle transports, and directional flow control (*45*, *46*) (see the representatives in fig. S22). Meanwhile, upscaling the designs to the meter scale could also find potential applications in temporarily deployable buildings (fig. S10B).

## MATERIALS AND METHODS

### Fabrication and mechanical test of the mechanical metastructure

The cube-shaped structural elements (overall dimension of 2 cm by 2 cm by 2 cm and shell thickness of 1 mm) of the proposed mechanical metastructure are printed by PolyJet 3D printing (Connex Objet 260, Stratasys). The rigid VeroWhite material (~0.68GPa) is used to print the rigid cubes. The structural elements are then connected using the ultra-adhesive plastic tape (Scotch, 4198W-SIOC) that acts as elastic hinges to create the assembled periodic mechanical metastructure. To enable the bistable deformation in the building blocks, strong fiber-enhanced ultra-adhesive tapes (Fiberglass Reinforced Tape, BOMEI PACK) are used to facilitate the feasible stretching of the mechanical metastructure into the deformed structural state before bifurcation, and then maintaining the stretched state with pins by fixing the fiber tapes onto the substrate. The repeating redundant structural parts are merged among the unit cells to save materials during fabrication of the tessellated metastructures, which, however, do not affect their reconfiguration behaviors. The bistable deformation feature of the structural unit is tested under uniaxial compression of the pop-up stable state 2 using Instron 5945 (see more details in the Supplementary Materials).

### Magnetic actuation of the bistable deformation of structural units

Thin-plate rectangular neodymium magnets (McMaster, 7048T29) with dimensions of 3 cm (length) by 1 cm (width) by 0.2 cm (thickness) are attached to the central parts of the structural units using double-sided tapes (Scotch Double-Sided Tape, 137DM-2). A permanent cylindrical neodymium magnet with diameter of 10 cm (McMaster, 5862K331) is used to remotely actuate the bistable switch in the local structural units.

### Experimental setups for the information encryption application

We developed a sensing and demonstration platform to visualize the SIE performance. We considered each pop-up strut as one single vertex and encoded the encrypted information onto the uniquely patterned pop-up struts in the form of vertices, lines, and/or polygons. To distinguish the “Information” (right diagonal triangle) and “Encryption” (left diagonal triangle) states, we assembled two adjustable infrared obstacle avoidance sensor modules (HiLetgo Inc.) beneath the left and right bottom vertices, respectively. Each of the modules contains a pair of infrared transmitting and receiving sensors that can measure the distance between the module and the vertex, which is used to identify the bistable state of the pop-up strut. After the structure pop-up, the distance between the sensor and strut becomes larger than the pre-adjusted threshold (30 mm) and triggers the sensor output signal change from “low” (0v) to “high” (5v). An Arduino Uno microcontroller (Arduino, Italy) is used to capture the sensors’ output signal in real time. When the microcontroller detected a binary logic change of these two signals, it printed “Encryption” or “Information” to the serial monitor in Arduino IDE.

## Supplementary Materials

### This PDF file includes:

Figs. S1 to S22 Tables S1 and S2 Notes S1 to S9 Legends for movies S1 to S9
