# Supplementary material for: Reprogrammable and reconfigurable mechanical computing metastructures with stable and high-density memory
Source: Sci Adv. 2024 Jun 26;10(26):eado6476. doi: 10.1126/sciadv.ado6476 (PMC11204216; doi:10.1126/sciadv.ado6476)
Supplement: Supplementary file 1 — Figs. S1 to S22 Tables S1 and S2 Notes S1 to S9 Legends for movies S1 to S9 [file sciadv.ado6476_sm.pdf]

Science Advances
AAAS

Supplementary Materials for

Reprogrammable and reconfigurable mechanical computing metastructures with stable and high-density memory

Yanbin Li *et al.*

Corresponding author: Hao Su, hsu4@ncsu.edu; Jie Yin, jyin8@ncsu.edu

Sci. Adv. **10**, eado6476 (2024)
DOI: 10.1126/sciadv.ado6476

The PDF file includes:

Note S1 to S9
Figs. S1 to S22
Tables S1 and S2
Legends for movies S1 to S9

Other Supplementary Material for this manuscript includes the following:

Movies S1 to S9

Supplementary text

S1. Summary of the comparison between conventional and unconventional mechanical computing systems

Table S1 and Table S2 summarize the comparison of the conventional and unconventional mechanical computing systems in terms of structural forms and materials compositions of mechanical components, actuation, and performances.

	**Old-generation mechanical computer**	**Recent mechanical computing systems**
**Types**	1. Mechanical clock;<br>2. Mechanical calculators (adding, multiplication, differentiation, polynomials analysis, etc.);<br>3. Mechanical counters;<br>4. Analogue computer (model and simulate)	1. Non-volatile systems with quasi-static equilibrium states (bistable/multi-stable deformations);<br>2. Volatile dynamic systems
**Structural form**	Three-dimensional (3D) bulky volume	1. 1D chain/array;<br>2. 2D Lattice and networks;<br>3. 3D periodic/aperiodic volumetric form
**Structural element**	Linkages;<br>gears	1. Deformable structures:<br>Beam, plate, shell, origami, kirigami etc.;<br>2. Material related: responsive to thermal, optical, chemical, magnetic, electric and their coupled stimulus
**Deformation mechanism**	Rotation and/or translation	1. Bending;<br>2. Snap-through buckling;<br>3. Twisting;<br>4. Vibration
**Underlying physics**	Simple kinematics	Nonlinear mechanics and dynamics;<br>Multiphysics problem
**Functionality**	Mathematical calculating;<br>Storing data;	Storing data;<br>Information processing;<br>Information transmission;<br>Robotic performances (imitating embodied control system, logical object manipulation and locomotion)
**Interaction with external environments**	No	Yes
**Programmability**	No	Yes

Table S1: **Comparison between the old-generation and recent mechanical computing systems.**

The mechanical components in conventional mechanical computing systems often utilize rigid links, levers, and gears as mechanical components that move by means of simple rigid rotation and translation driven by mechanical loading for performing calculations (Table S1). They are often very bulky.

			Structural element	Deformation form	Materials	Stimuli	Mechanical computing performance
Previous works	1D	[3]	Kresling origami	Twisting	Magnetic polymer	Magnetic field	Information storage
		[4]	Curved beam	Snap-through buckling	Magnetic polymer	Magnetic field	Information transmission<br>Binary information computing
		[10]	Thin shell	Shell buckling	Ecoflex	Mechanical load	Binary information computing;<br>Controllable robotic valve
		[20]	Beam network	Beam deflection	Rubber like polymer	Mechanical stretching	Binary information computing
		[26]	Truss	Beam deflection	PLA (Polyester)	Compression	Binary information computing
		[30]	Kresling origami	Twisting	Wood paper	Mechanical vibration	Information storage
	2D	[5]	Thin shell	Shell inflation	Liquid crystal elastomer	Mechanical inflation	Information display
		[11]	Rhombic rotatable unit	Beam bending	Ecoflex	Mechanical compression	Binary information computing
		[12]	Rhombic rotatable unit	Beam bending	Ecoflex	Mechanical compression	Information encryption and perception
		[17]	Thin dome	Shell buckling	Flexible polymer	Mechanical compression	Binary information computing
		[23]	Cube frame<br>dome shell	Beam bending<br>and dome shell buckling	Magnetic polymer<br>and ecoflex	Magnetic field	Information storage<br>Information display
		[21]	Beam network	Beam buckling	PLA	Mechanical compression	Sequenced binary information computing;<br>Mechanical logic for MEMS;<br>Information transmission
		[24]	Lattice network	Beam vibration	Acrylic	Mechanical vibration	Information display;<br>Information encryption;<br>Mechanical calculating
		[25]	Lattice network	Beam bending	PLA	Mechanical compression	Information learning;<br>Information memorizing;<br>Information sensing: interact with external environment
	3D	[29]	Waterbomb origami	Origami folding	Plastic thin film	Mechanical compression	Binary information computing
		[2]	Clamped beam	Beam buckling	Hydrogel (humidity)	Humidity	Binary information computing
		[7]	Thick plate	Compression	Liquid metal and PDMS with pigment	Electric current	Interactive device
		[15]	Cubic frame	Beam bending	Ecoflex	Mechanical compression	Information encryption
		[31]	Rhombic kirigami unit	Beam bending	TPU, PLA, and PETG	Mechanical stretching	Information storage;<br>Information display;
		[36]	Cylindrical curved shell	Shell buckling	PLA	Mechanical compression	Information storage
**Our work**			Thick origami network	Kinematic bifurcation enabled snap-through instability	PLA	Magnetic field	Information storage;<br>Binary information computing;<br>Information display;<br>Information perception and encryption;<br>Information processing beyond binary bits

Table S2: **Comparison between the previous works and our work on mechanical computing systems.**

In contrast, recent advances in unconventional mechanical computing utilize lightweight, deformable, and flexible mechanical components made of various non-stimuli- and stimuli-responsive materials for information processing and storage (Table S1-S2). Differently, information can be encoded in its deformed configurations, materials properties, and structural

forms. The structural forms range from 1D chain and array and 2D lattice and network to 3D periodic and non-periodic volumetric architectures. Specifically, the structural elements take the forms of curved beam, truss, curved thin shells, plates, beam networks, origami/kirigami structures, and architected materials. They are often made of various soft polymers and elastomers, which allow them for actuation under mechanical loading or external stimuli such as light, heat, humidity, electricity, and magnetic fields (Table S2). Correspondingly, the deformation modes are rich, including stretching, compression, bending, twisting, folding, and buckling (Table S2). In terms of performances, the unconventional mechanical computing systems enable more functionalities, including but not limited to information storage, information transmission, binary information computing, information display, information encryption and perception, mechanical computing, memory, learning, and sensing (Table S2).

For unconventional mechanical computing systems, previous works are (i) limited to binary information, (ii) challenging to be reprogrammed by transforming its original shape beyond binary state change, and (iii) susceptible to mechanical perturbation for preserving stored information. In contrast, our work can be programmed to be binary and reprogrammed to be beyond binary via shape reconfiguration based on the coupled kinematics and mechanics in a new class of reconfigurable, deformable mechanisms. It shows stable mechanical memory even under external perturbations. In terms of the performance, previous works are limited to one or few mechanical computing functionalities such as information storage, display, computing, and encryption (Table S2). However, ours can perform multi-functional capabilities using the same designed system.

S2. Combinatorial design of the hierarchical building blocks

Given the unique structural features of our selected simple cube-shaped structural components, we note that both the hierarchical level-1 and level-2 structural units can be combinatorially constructed by randomly arranging the 4R looped-linkage-based hinges. As shown in **fig. S2A**, a total of four cube-side positions can be used to place one of the 4R hinges. Therefore, we can obtain 44 different designs for the level-1 structural units. Among them, there are only 58 different designs with one structural mobility (or degree of freedom-DOF) and 4 designs with 2 structural mobilities (**fig. S2B**). For the reconfigurable level-1 structural units, two different types of reconfiguration exist for the design cases with only one DOF, i.e. 1D chain-like (**fig. S2C(ii)**) or

in-plane rotation (**fig. S2C**(**iii**)). Meanwhile, there are two different directional chain-like reconfigurations for the level-1 building block with two DOFs (**fig. S2C**(**i**)).

Similarly, the combinatorial design concept can be applied to the 4R loop-linkage arrangement of level-2 structures. Interestingly, we find that when selecting the level-1building block as its four links placed and connected symmetrically, the level-2 structure always shows mobility, see the design representatives in **fig. S3**. Therefore, for simplicity, we select the optimal design, the unit type 2, which can be reconfigured with out-of-plane extrusions. Compared to other level-2 structural units, the unit type 2 can easily and compatibly reconfigure with both chain-like path and out-of-plane extrusion path, which favor both the combinatorial reconfigurations and bi-stable deformations as demonstrated in **Figs. 3 and 4**.

S3. Reconfiguration kinematics of the level-2 building block

Given the looped-linkage-mechanism structure feature, the reconfiguration kinematics and the kinematic bifurcation of level-2 structural unit can be analyzed based on the Denavit-Hartbenberg theorem by building the local coordinate systems in **fig. S4**.

For the level-2 structural unit in **Fig. 2**, the local coordinate systems are built in **fig. S5**. Then, we can use a transformation matrix **M** to describe the relative geometrical relations among all the level-2 hinges. Specially, the transformation matrix **M** takes the form as

$$\left[\boldsymbol{M}_{i(i+1)}\right]=\begin{bmatrix} 1 & 0 & 0 & 0 \\ -a_{i(i+1)} & \cos\theta_i & \sin\theta_i & 0 \\ -s_i\sin\alpha_{i(i+1)} & -\cos\alpha_{i(i+1)}\sin\theta_i & \cos\alpha_{i(i+1)}\cos\theta_i & \sin\alpha_{i(i+1)} \\ -s_i\cos\alpha_{i(i+1)} & \sin\alpha_{i(i+1)}\sin\theta_i & -\sin\alpha_{i(i+1)}\cos\theta_i & \cos\alpha_{i(i+1)} \end{bmatrix} \tag{S1}$$

wherein $\alpha_{i(i+1)}$ is the angle between the successive hinge axis z_i and hinge axis z_{i+1} positively with right hand rules in the positive direction of axis x_{i+1}; $a_{i(i+1)}$ is the perpendicular distance between the hinge axis z_i and hinge axis z_{i+1}; $\theta_{i(i+1)}$ is the angle between the link bar axis of *i*(*i*+1), i.e., x_i and the link axis of *i*(*i*+1), i.e., x_{i+1}, positively along the positive direction of axis z_i with right hand rule; $s_{i(i+1)}$ the perpendicular distance between the *x*-axes.

For the level-2 building block with cuboid-shaped structural components and dimensions of *l* × *h* × *w* (Supplementary fig. S4A), we have

$$
\begin{aligned}
&a_{12} = a_{23} = a_{34} = a_{41} = \begin{cases} h\cos\theta_1 - l\sin\theta_1 & 0 \le \theta_y \le tan^{-1}(h/l) \\ l\sin\theta_1 - h\cos\theta_1 & tan^{-1}(h/l) \le \theta_y \le 90^\circ \end{cases}; \\
&s_{12} = s_{34} = h\sin\theta_1 + w\cos\theta_2 - w; \\
&s_{23} = s_{41} = h\sin\theta_1 + l\cos\theta_1 - l;
\end{aligned} \tag{S2}
$$

Thus, for the level-2 building block in **Fig. 2** with $l = h = w$, Eq. (S2) can be rewritten as

$$
\begin{aligned}
&a_{12} = a_{23} = a_{34} = a_{41} = \begin{cases} \cos\theta_1 - \sin\theta_1 & 0^\circ \le \theta_y \le 45^\circ \\ \sin\theta_1 - \cos\theta_1 & 45^\circ \le \theta_y \le 90^\circ \end{cases} \\
&s_{12} = s_{34} = \sin\theta_2 + \cos\theta_2 - 1 \\
&s_{23} = s_{41} = \sin\theta_1 + \cos\theta_1 - 1
\end{aligned} \tag{S3}
$$

Based on the Denavit-Hartbenberg theorem for the cube-shape based level-2 building block, its reconfiguration kinematics can be derived according to

$$\prod_{n=1}^{4} \mathbf{M}_n = \mathbf{I} \tag{S4}$$

and

$$\theta_1 - \theta_2 \equiv 0 \tag{S5}$$

i.e., the reconfiguration paths 2 and 3 shown in **Fig. 2**, ii.

Moreover, based on equations (S1) and (S3-S5), by using the singular value decomposition method, the kinematic bifurcation of the cube-shaped level-2 building block can be determined. With an infinitesimal increment on the opening angle θ_k by $\delta\theta_k$, (k is integer with k=1, 2, 3 and 4), we can rewrite equation (S4) as

$$\left[\boldsymbol{M}_k^{\theta_k+\delta\theta_k}\right] = [\boldsymbol{M}_k^{\theta_k}] + [\boldsymbol{M}_k^{\theta_k}]' \delta\theta_k \tag{S6}$$

where $\mathbf{M}^{\theta}$ is the rotation part decomposed from transformation **M**, i.e. $\mathbf{M}=\mathbf{M}^{\theta}\mathbf{M}^{\mathrm{T}}$ with $\mathbf{M}^{\mathrm{T}}$ being the translational part

$$[\boldsymbol{M}_k^{\theta_k}]' = \left[\frac{\partial \boldsymbol{M}_k^{\theta_k}}{\partial\theta_k}\right] = \begin{bmatrix} -\sin\theta_k & \cos\theta_k & 0 & 0 \\ -\cos\theta_k & -\sin\theta_k & 0 & 0 \\ 0 & 0 & 1 & 0 \\ 0 & 0 & 0 & 1 \end{bmatrix} \tag{S7}$$

Combining equations (S6) and equation (S4), we can get a coefficient matrix C from

$$\sum_{k=1}^{4} \boldsymbol{C}_k \delta\theta_k^2 = \left(\prod_{k=1}^{4} \boldsymbol{M}_k^L [\boldsymbol{M}_k^{\theta_k^2}]'\right)\delta\theta_1^2 + \cdots + \left(\prod_{k=1}^{4} \boldsymbol{M}_k^L [\boldsymbol{M}_k^{\theta_k^2}]'\right)\delta\theta_4^2 \tag{S8}$$

Thus, the singular value decomposition method can be used with

$$\mathbf{C} = \mathbf{UVW}^{\mathrm{T}} \quad \text{(S9)}$$

wherein for the level-2 building block, **U** and **W** are the two 4×4 and 4×4 square orthogonal matrices containing left- and right-singular vectors, respectively, while **V** is a 4×4 matrix with I non-zero singular values vii on its main diagonal and I is the rank of the matrix **C**. Then, according to the results of whether the singular value vii is equal to zero, we can determine the occurrence of kinematic bifurcation. The results are shown in **fig. S6**. It shows that kinematic bifurcation can always occur on the reconfiguration paths 2 and 3.

Moreover, based on equation (S5), we can simply calculate the in-plane expansion change W of the cube-shaped level-2 building block and its out-of-plane extrusion H as

$$\begin{aligned} H &= l\left(1+\sin\theta_1 - \cos\theta_1\right) \\ W &= l(3+\cos\theta_1 + \sin\theta_1) \end{aligned} \quad \text{(S10)}$$

with the results shown in **Fig. 2C**.

S4. Experimental test of the bistable building block

As shown in **fig. S8**, the bi-stable deformation of our building block can only be achieved for a pre-tensioned unit. To experimentally validate the structural bi-stability, we firstly stretch the unit and then fix it with a double-sided adhesive tape on a substrate (**fig. S8A**). We experimentally realize the fixed boundary by pinning the metal pins on ultra-adhesive fiber tapes under the pre-stretched state. We use the displacement-control method to uni-axially compress the pulled-up samples to verify their bi-stable deformation feature. Double-sided tape is used to bond the sample onto a compression holder made of an acrylic plate. The sample is fixed on a hard plate holder with metal pins. Specially, to avoid the out-of-plane extrusionof the boundary structural part, we conduct the uniaxial compression (by Instron) firstly on the sample at its second stale state (**fig. S8B**) to further bypass the bifurcation point with θc=45º. Several intermediate structural states under different compression strains withε = 0, 4%, 33% and 90% are displayed in **fig. S8C**. From the testing results shown in **fig. S8D**, we can see the bi-stable structural deformation behavior induced by the snap-through instability with negative force area.

S5. Kinematics changes induced bistability

The kinematic change resulted in contradiction when the building block undergoes bistable deformation. Intrinsically, it is induced after introducing structural constraints by partially fixing the boundary of the building block. In the following, we elaborate this with more specific details.

As illustrated in **Fig. 4B**, our proposed building block need to be first stretched to a configuration before the bifurcated state. It should note that this reconfiguration process is completely *kinematic compatible*. Mathematically, it can be described based on the defined central and corner opening angles as

$$\theta_c = \theta_y = \theta_0 \tag{S11}$$

Note that θ_0 must be smaller than 45° to guarantee the occurrence of bistable deformation in the building block.

Then, as shown in **Fig. 4B** (middle), we introduce structural constraint at the boundaries of the building block to induce bistable deformation, i.e., the constrained stable state 1. Specially, the boundaries of the building block is not completely constrained. The building block can rotate out of plane. Therefore, with the out-of-plane deformation freedom under the in-plane constraints, the building block can deform into another constrained stable state 2 by pulling up the central structural parts (**Fig. 4B**, right).

Specifically, during the state transition process from constrained stable state 1 to stable state 2, kinematics will change and be different from the original compatible reconfiguration kinematics. Thus, kinematic contraction will occur for the rotations of the central and corner folds, which mathematically can be expressed as

$$\theta_c \neq \theta_y \tag{S12}$$

This kinematic contraction always exist during the bistable deformation process until the building block reaches its second constrained stable state.

To more clearly explain this kinematic contradiction, we add one more supporting figure (**fig. S9**) by showing the intermediate reconfiguration of the constrained building block. As illustrated in **fig. S9**, we present the *free body diagrams* of the building block at the cross section bypassing the boundary constrains (**fig. S9A** and **S9B**). Given the out-of-plane rotation freedom, we can simplify the constrained boundaries of the building block as fixed hinges. Therefore, the building block cannot shrink or expand in plane (i.e., the x-y plane as defined in **fig. S9**), and structural parts close to the boundaries can only rotate out of plane. Thus, when the building block is vertically pulled (*z* direction defined as **fig. S9**) to transit from constrained stable state 1 to constrained stable state

2, the boundary structural parts have to rotate out of plane to offset the in-plane expansion of the building block to make the whole constrained building block deformable. Particularly, we assume the boundary structural parts rotate with angle as θ_b. We know that θ_b increases from zero to its maximum first and then decrease to zero.

Meanwhile, the stretched structural part is always being pulled up along the *z* direction until the disappearance of the kinematic contradiction with θ_b reducing to zero. Thus, at the second constrained stable state, we have

$$\theta_b = 0^o,\ \theta_c + \theta_y = 90^o \quad \text{(S13)}$$

For the deformed structural profile, when pulling up the central structural part, the in-plane expansion reconfigurations (**Fig. 2C**) is always suppressed by the boundary constraints and transformed into the out-of-plane rotation of close-to-boundary structural parts. Thus, energy barrier will show up due to this kinematic contradiction, inducing structural bistability in the constrained building block. Note that similar deformation process can occur when the constrained building block reversibly deforms from the second constrained stable state to the first constrained stable state.

To conclude, from the above analysis and **fig. S9**, the deformed profiles of the constrained building blocks are theoretically allowable but exhibiting new deformed features shown in the middle of **fig. S9C**. Truthfully, the hinges keep undergoing simple rotational deformation while the constrained boundary conditions remain.

S6. Reconfiguration into pyramid-like architectured structures

The multi-layered pyramid shape shown in **Fig. 6E-6F** is based on a sequential reconfiguration process. As shown in **Fig. 6F**, we need firstly pre-stretch a 2 × 2 mechanical metastructure and then extrude the central local structural element to its second stable state. Then, we will compress the bi-stably deformed mechanical metastructure to a compact form. By directly and vertically stretching the extruded central structural element, we can obtain the final pyramid shape. The whole process is shown in **fig. S10A**.

For the metastructure with more unit cells, a similar sequential reconfiguration process can be applied to achieve a higher pyramid-like architecture **(fig. S10B)**. Particularly, for a metastructure composed of k × k unit cells, the structural height *h* and the enclosed internal volume V_k of its transformed pyramid shape can be simply calculated as

$$
\begin{aligned}
h &= 2kl + 1 \\
V_k &= l(3 + \cos\theta_1 + \sin\theta_1)
\end{aligned} \tag{S14}
$$

Based on the pulling method, we can achieve more reconfigured configurations with periodic hierarchical multi-layered local structural segments shown in **Fig. 6F** and **figs. S10C-10D**, which can be used as information bits. Thus, our structure can have higher information density compared to previous works given the additionally generated independently deformable local structural segments (**Fig. 6C**) and multi-layered structural patterns (**Figs. 6E-6F** and **figs. S10C-S10D**).

The relationship between the information states and unit cell density can be given by

$$
\begin{aligned}
m_2 &= (N/4n)^2 + (N/4n-2)^2 \quad n = 2 \\
m_n &= (N/4n)^2 \quad n = 2k, k = 2, 3 \ldots
\end{aligned} \tag{S15}
$$

where n is the information state with $n = 2k$ (k is a positive integer), N is the total number of the cube-shaped elements along one side of the metastructure, and m_n is the number of unit cells corresponding to information state n, representing the unit cell density.

Therefore, for our proposed mechanical meta-structure with N cube-shaped structural elements along one side, we can define $\overline{m}$ to straightforwardly compare the unit cell density corresponding to different information states with

$$
\overline{m} = (N/4n)^2/[(N/8)^2 + (N/8-2)^2] \quad n = 2k, k = 2, 3 \ldots \tag{S16}
$$

Based on the above equation, we can see the decrement of the unit cell density when the information states rise to higher levels (**fig. S10E** with $N = 96$).

Outperforming previous designed mechanical metamaterials/metastructures based information systems which can only store binary information, our proposed mechanical metastructure can store more information under the same quantity of information units and thus exhibit higher information density. To demonstrate it, considering a 2D platform with m × m building blocks, we can calculate the total number of information units in our designed structure as

$$
n_{total} = n_{binary} + n_{higher\text{-}level} \tag{S17}
$$

where n_{binary} is the number of information units for binary information storage and $n_{higher\text{-}level}$ is the higher level information storage. We plot the comparison results between our structure and previous binary information systems (**fig. S10E**). The information units of our structure significantly outnumber the previous designs and thus exhibit high information density. The information storage capacity increases with the number of building blocks.

S7. Experimental test of the untethered magnetic actuation

We have demonstrated that the bi-stable deformation of the local structural elements can be easily actuated through permanent magnets. As shown in **fig. S14A**(**i**), a thin-plate shaped passive rare-earth magnet (6cm×1cm×0.2cm) is attached onto the top surface of the local structural elements by using double-sided ultra-adhesive tape (Scotch). A strong Rubidium magnet (**fig. S14A**(**ii**)) is used to actively trigger the bi-stable deformation of local structural elements by "attracting" the attached passive magnets.

The extruded structural elements are expected to be not affected by the active magnets when actuating their adjacent non-extruded structural elements. To examine it, we measured the magnetic field intensity distribution of both the passive and active magnets using the magnetometer (**fig. S14B**). As shown in **fig. S14C,** the magnetic field distribution $\mathbf{B}_{z1}$ of the passive magnet will sharply die down along its thickness direction in a very short distance. For example, the strength of $\mathbf{B}_{z1}$ can suddenly drop from ~ 4mT at the position z_1=0.2cm to about 0.65 mT at the position z_1=2cm. For the active magnet, we monitored its magnetic fields along two directions: the vertical direction and the radial direction. Compared with the passive magnet, the strength of the magnetic field $\mathbf{B}_{z2}$ of the active magnet will significantly drop from 12mT at z2=10cm to about 3.3mT at z2=15cm (**fig. S14D**), which, however, is still significantly larger than the strength of $\mathbf{B}_{z1}$ at the same testing distance (over 100 times). Interestingly, we find the strength of $\mathbf{B}_{R2}$ (i.e., along the radial direction of the active magnet) will drop to almost zero beyond the distance between two local structural elements about 12 cm.

Therefore, we can conclude that the pulling force is mainly induced by the active magnet, while the passive magnet is to guarantee the pulling-up of local structural elements, and the extruded structural elements at their second stable states will not be influenced by the active magnets when it actuates the un-deformed the surrounding structural elements at their 1st stable states. Moreover, we also found that for the 3×3 metastructure (**fig. S15A**), the pulling force of the central structural element shows the maximum value, which is attributed to the stronger boundary constraints compared to its surrounding eight local structural elements (see **fig. S15**).

More significantly, we observe that the extruded local structural elements can stably maintain their second stable states when further pulling-up or pushing-down their surrounding bi-stable structural elements repeatedly. This stability robustness arises from the slightly variation of the opening angle θ_c. As shown in **fig. S15A**, we verify this phenomenon by monitoring the changes of the

opening angle of the central structural element θ_c when bi-stably deform its adjacent structural element with relative coordinates of (2, 1) at θ_c = 20º. Indicated by the results shown in **fig. S15B**, we find that θ_c just slightly increases with a change of $d\theta_c \leq \approx 0.7º$. Given the singular opening angle for kinematic bifurcation at 45º, local structural elements can be bi-stably deformed not only independently without influencing to each other but also robustly capable of maintaining their extruded 2nd stable states under an appropriate pre-stretching.

S8. Design details of the mechanical binary logic gate

The input systems are based on three local structural elements with relative positions shown in **fig. S17A**. Prim(s) with rectangular cross section shape and a quadrangular-shaped pyramid are attached on the top. The output systems include three prisms as the supporting columns enclosed with elastic rubber bans used for stable outputs, and a flat plate connected with the rubber band acting as output surfaces. The integrated system including input and output systems are shown in **fig. S17A**. We use the theorem of "one plane with one point" or "three different points" to form a stable 2D flat plane as the basis to perform "AND" and "OR" binary logic gates. The prism top flat surface can be treated as the "plane" while the vertex of the pyramid shape as a "point". The bi-stable extrusions of the local structural elements can translate their upper attached structural components surface or vertex point to new positions whose combinations can correspondingly move the output surface to new places or not. Finally, the binary "AND" and "OR" logic gate can be mechanically performed based on our built mechanical metastructure.

To perform 'OR' mechanical logic gate, as shown in **fig. S17B** (left and middle), two identical prisms (white color) and one quadrangular-shaped pyramid with the same structural height as the two prisms are pasted onto three local structural elements. By extruding one of the local structural elements with prisms and that with pyramid shapes, we can correspondingly extrude the output surface to the same spatial position, which thus can be acted as the (1,0) and (0,1) logic binary computation for "OR" gate. Moreover, the output surface can extend to the same position if extruding all the three structural elements to their 2nd stable states as shown in **fig. S17B** (right). In experiments, we treat the bi-stable extrusion of both the surface S_1 and point P_1 together as (1,0) type of input, the bi-stable extrusion of both the surface S_2 and point P_1 together as (0,1) type of input and all the surface S_1, S_2 and point P_1 as the (1,1) type of input.

To perform the “AND” mechanical logic gate, three identical pyramid shaped structures are added onto three different local structural elements shown in **fig. S17C**. Therefore, unless extruding all the three local structural elements as inputs to perform the (1,1) to “1” binary logic gate, all the other situations will not change the output position of the output surface shown in **fig. S17C** (left three, see the center of the output surface), i.e., the “AND” mechanical logic gates (0, 0), (0, 1) and (1, 0) to “0” output can be performed. We treat the bi-stable extrusion of the points $P_{2,3}$ together as (1,0) type of input, the bi-stable extrusion of point P_1 as (0,1) type of input and all the points $P_{1,2,3}$ together as the (1,1) type of input.

Particularly, we note the “AND” and “OR” logic gates rely only on several local structural segments. Therefore, given the deformation independence among different structural segments at the pre-stretched configuration, we demonstrate the parallel mechanical computation of our proposed mechanical metastructure. As shown in **fig. S18**, we can build multiple different binary logic gates to different local structural segments. Due to their deformation independence, we can perform parallel binary mechanical computation on a single platform, see the schematic matrix shown in **figs. S18A-S18B**, ii. We can also achieve “NAND” and “NOR” binary logic computation on our metastructure just by adding more unique structural components shown in **fig. S19**.

S9. Scalable and extended designs to thick rubber plates

It is challenging to scale down the proposed mechanical metastructure using the current bonding and 3D printing method. Since all the hinges are located on its top and bottom surfaces of the metastructure with no hinges along the thickness direction, it becomes feasible to fabricate similar metastructures using laser cutting. As demonstrated in **fig. S21**, a unit cell with much smaller dimensions (20mm × 20mm × 5mm) can be constructed from a thick rubber sheet via laser cutting. To create the folds at different surfaces (top and bottom surfaces shown as **fig. S21A**), we firstly fix the rubber plate in a coordinated system and cut the rubber plate in a non-cut-through way by controlling the laser energy (Energy: 50%, Speed: 50%, Frequency: 2000Hz with Laser-cutting machine Epilog Laser Mini 18) and 16 cutting time, and then flip it and perform the second cutting. Stretching the smaller sample leads to the pop-up of the structure, which shows similar bi-stable deformation features (**fig. S21B** and **S21C** and Movie S9).

Supplementary Figures S1 to S22

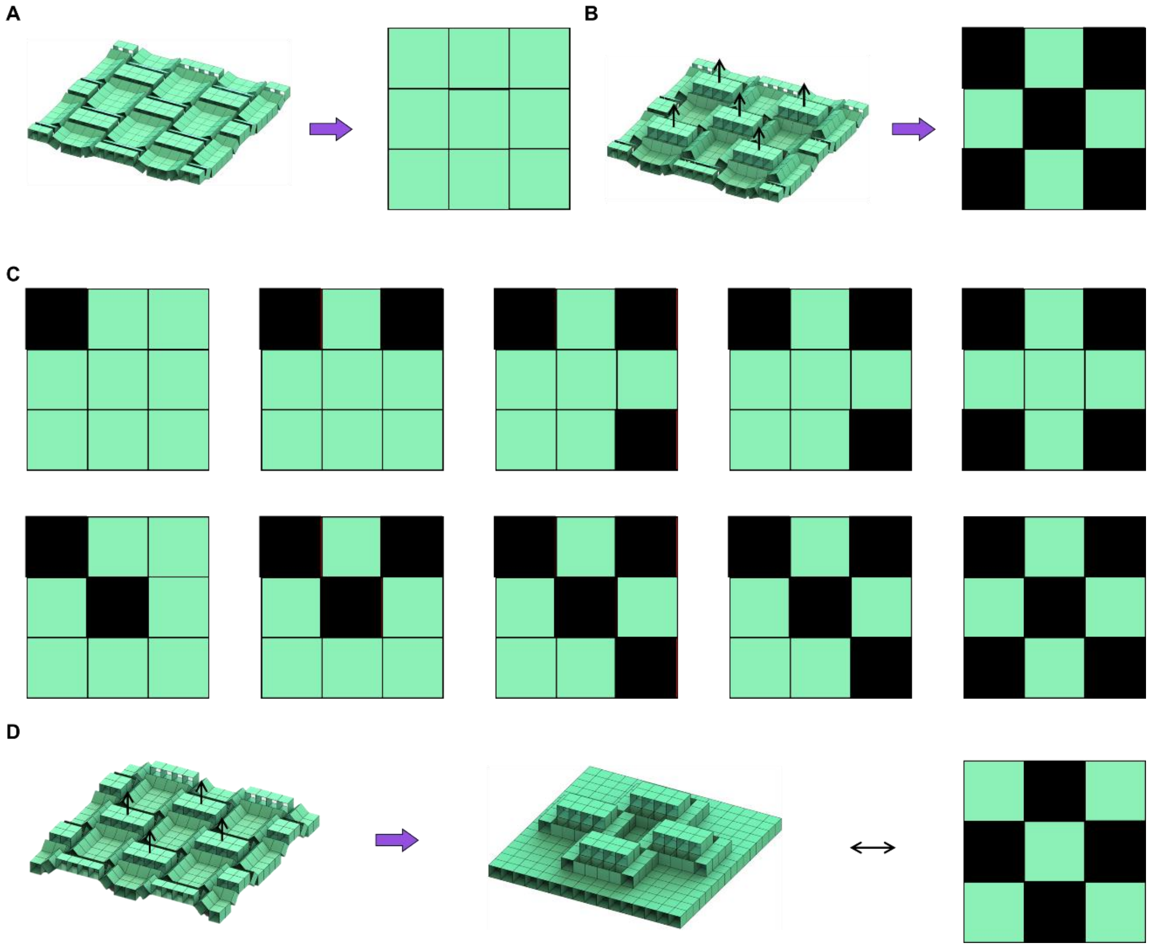

Supplementary Fig. S1. Combinatorial deformed patterns of the proposed mechanical metastructure shown in Fig. 1A. (**A**) Schematics of mapping the 2 by 2 deformed mechanical metastructure (before bifurcation) onto a 3 by 3 pixel matrix. (**B**) Schematics of one combinatorial deformed pattern of the mechanical metastructure at bifurcation state: black pixel as pop up part and green pixel as parts without pop up. (**C**) Other combinatorically deformed pixel patterns by selectively stretching up local structural elements. (**D**) Schematics process of reconfiguring the deformed pattern to the final pixel display configuration with structural stability by simple stretching.

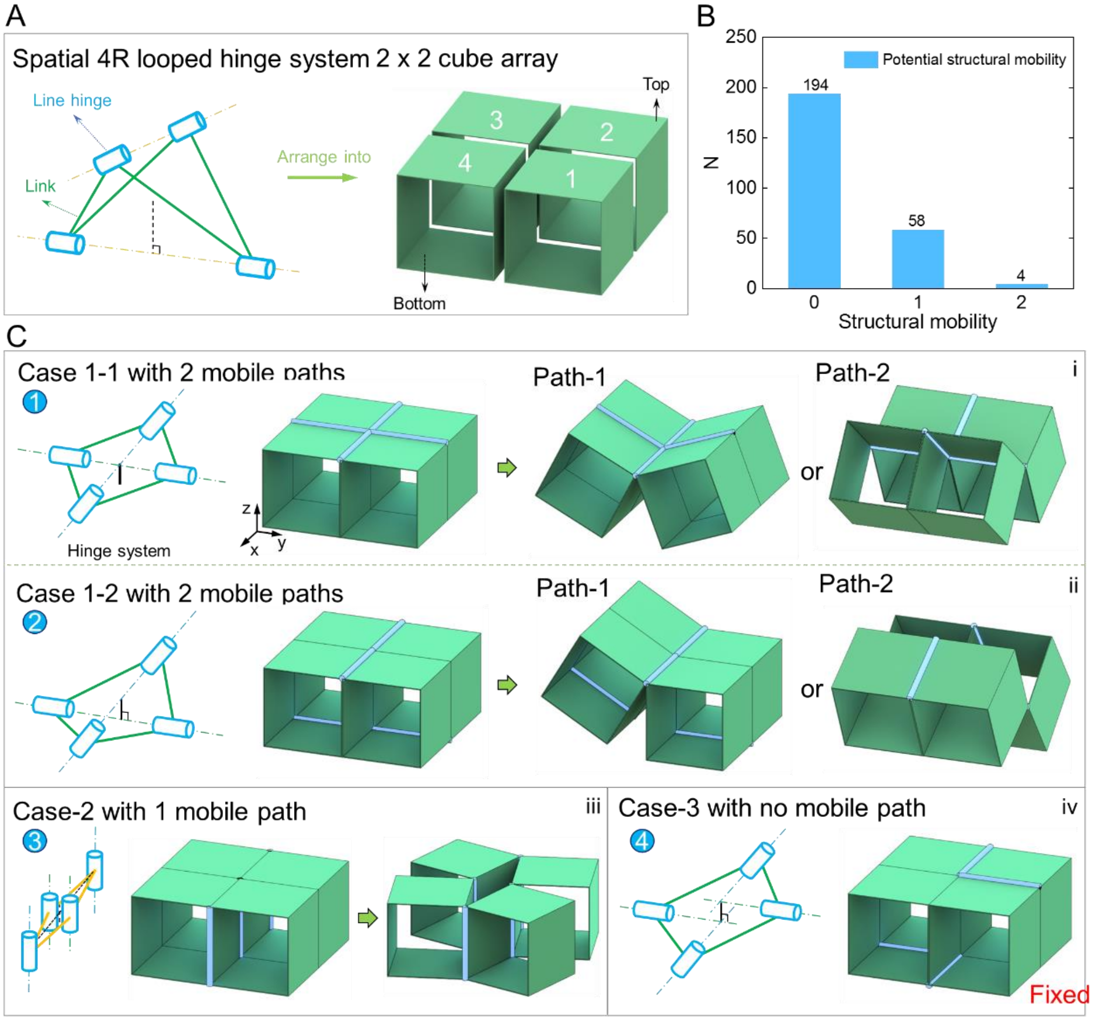

Supplementary Fig. S2. Combinatorial design of level-1 building block. (**A**) Schematic illustration of the design details of level-1 building block with 2 by 2 cube-shaped structural components connected with a 4R looped-linkage-mechanism. (**B**) The number of level-1 building block with 0, 1 and 2 structural mobilities. (**C**) Reconfiguration features of the level-1 building block with two (i), one (ii-iii) and zero (iv) structural mobility.

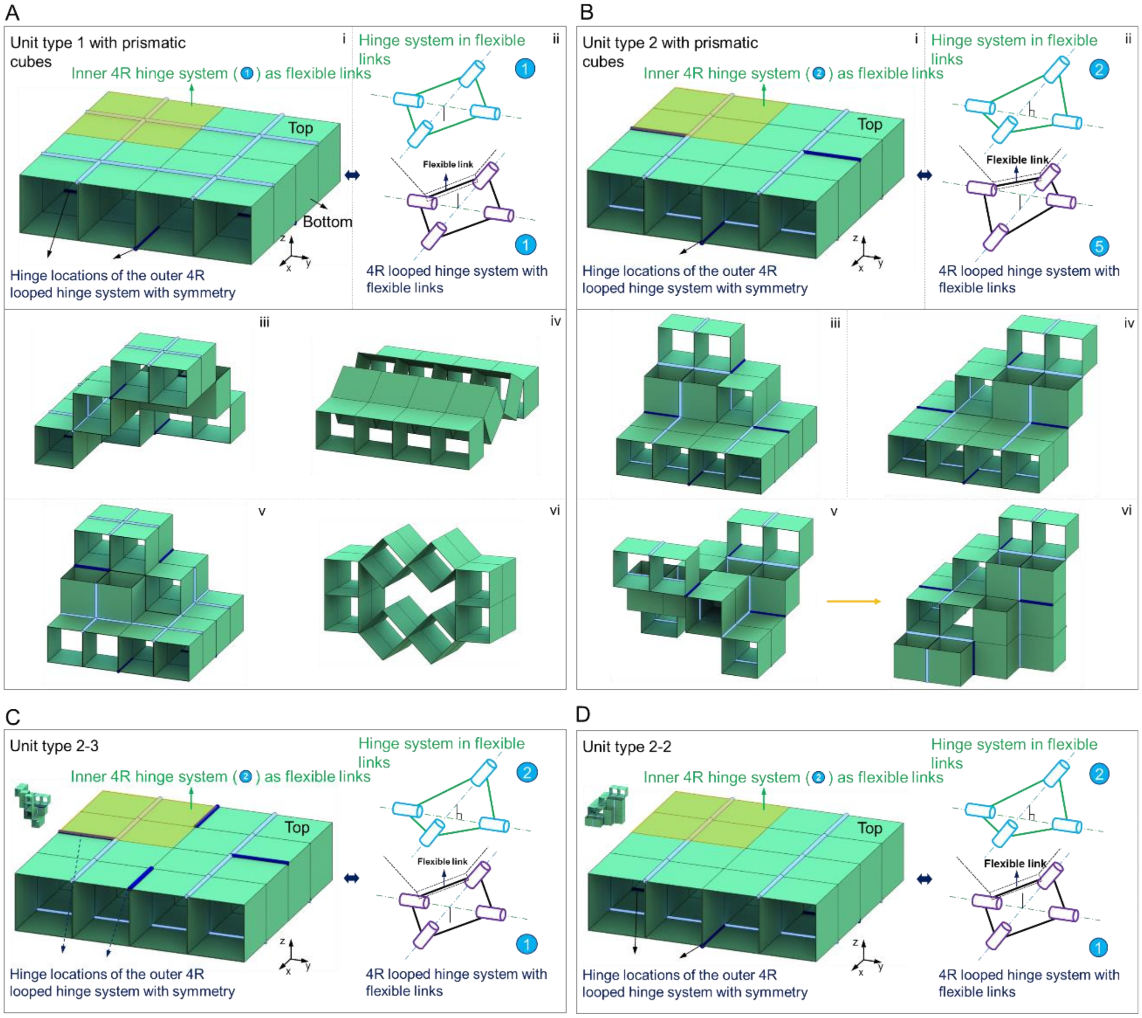

Supplementary Fig. S3. Combinatorial design of level-2 building block. (**A**) Schematic illustration of the design details of type 1 level-2 building block with 2 by 2 level-1 building block based structural components connected with a 4R looped-linkage-mechanism (i-ii) and its reconfiguration details (iii-iv). (**B**) Schematic illustration of the design details of type 2 level-2 building block (i-ii) and its reconfiguration details (ii-vi). (**C-D**) Schematic illustration of the design details of type 2-2 and 2-3 level-2 building blocks.

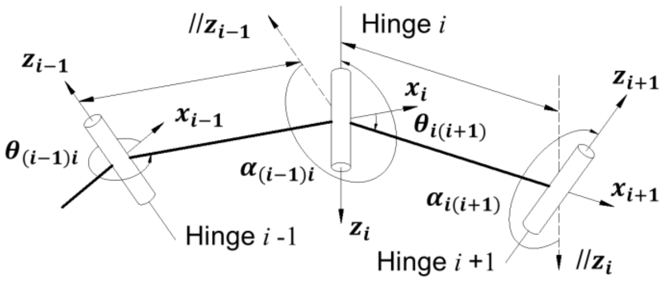

Supplementary Fig. S4. Illustration of the geometrical parameters definitions of consecutive local coordinate systems.

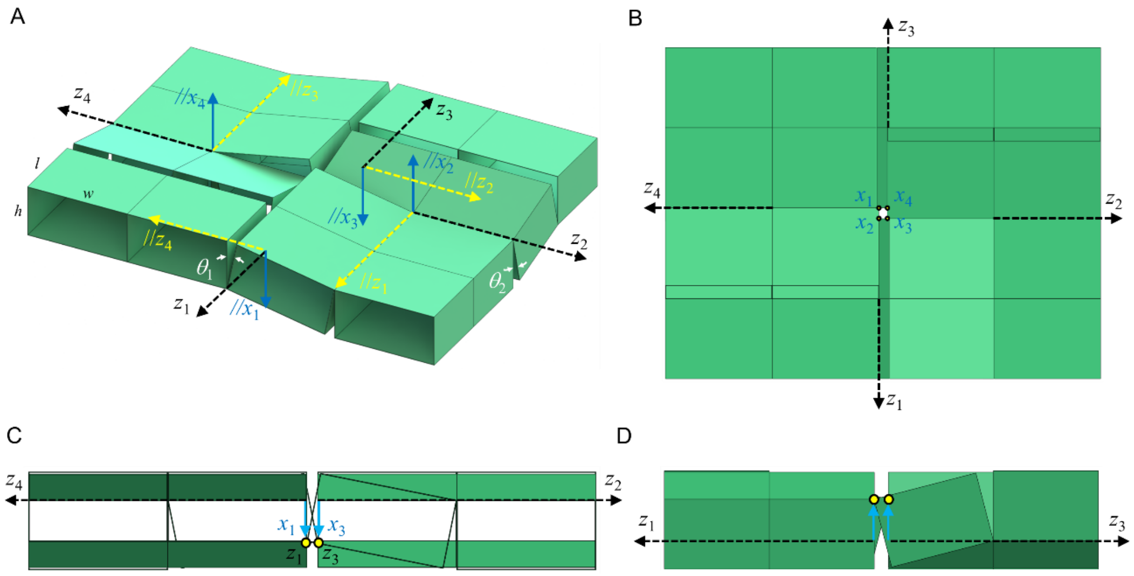

Supplementary Fig. S5. Schematic illustration of geometrical parameter definitions of the level-2 building block with arbitrary dimension. (**A**) Construction of local coordinate systems at the level-2 hinges. (**B**) Top view. (**C-D**) Side views.

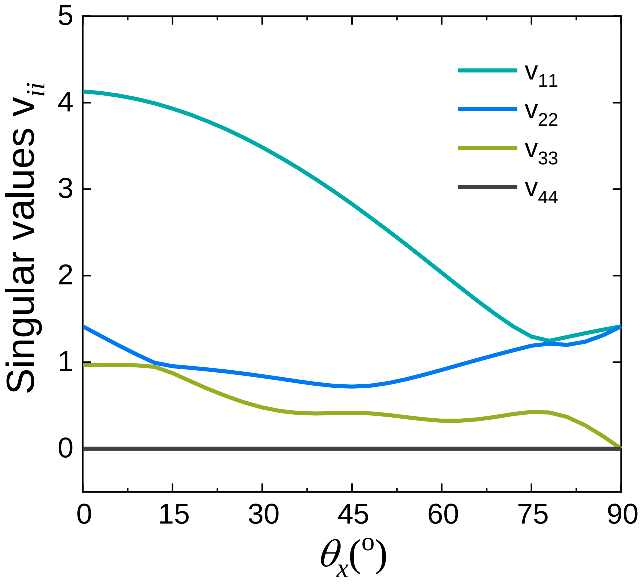

Supplementary Fig. S6. Variations of the four singular values.

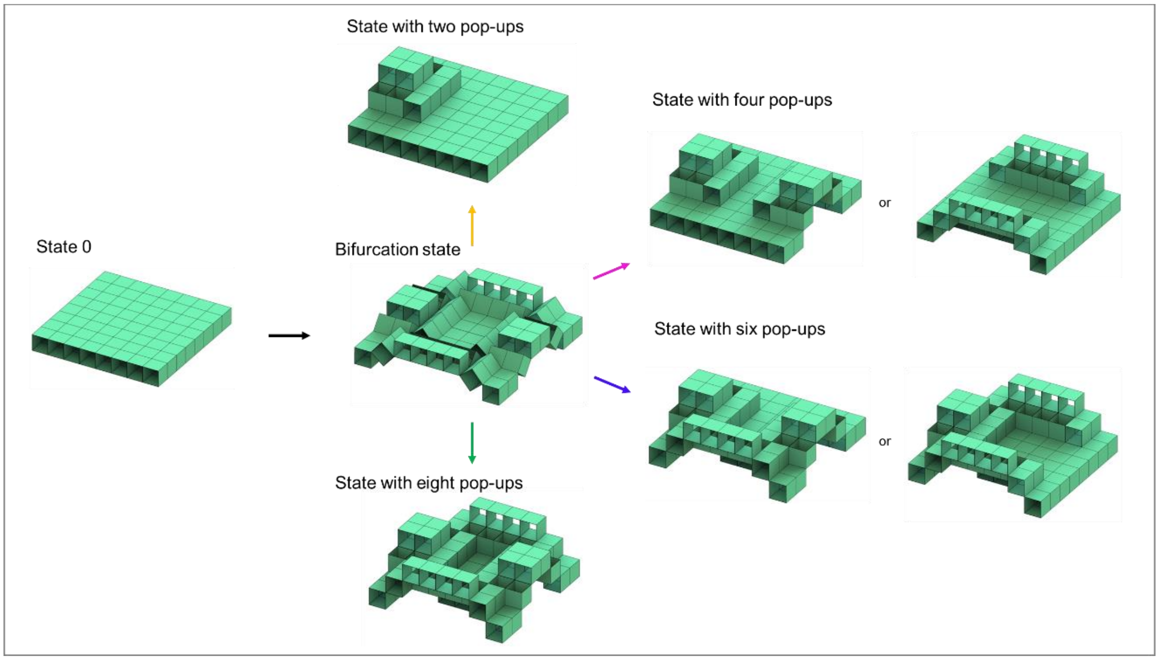

Supplementary Fig. S7. Schematic illustration the reconfiguration details of the unit cell when being flipped.

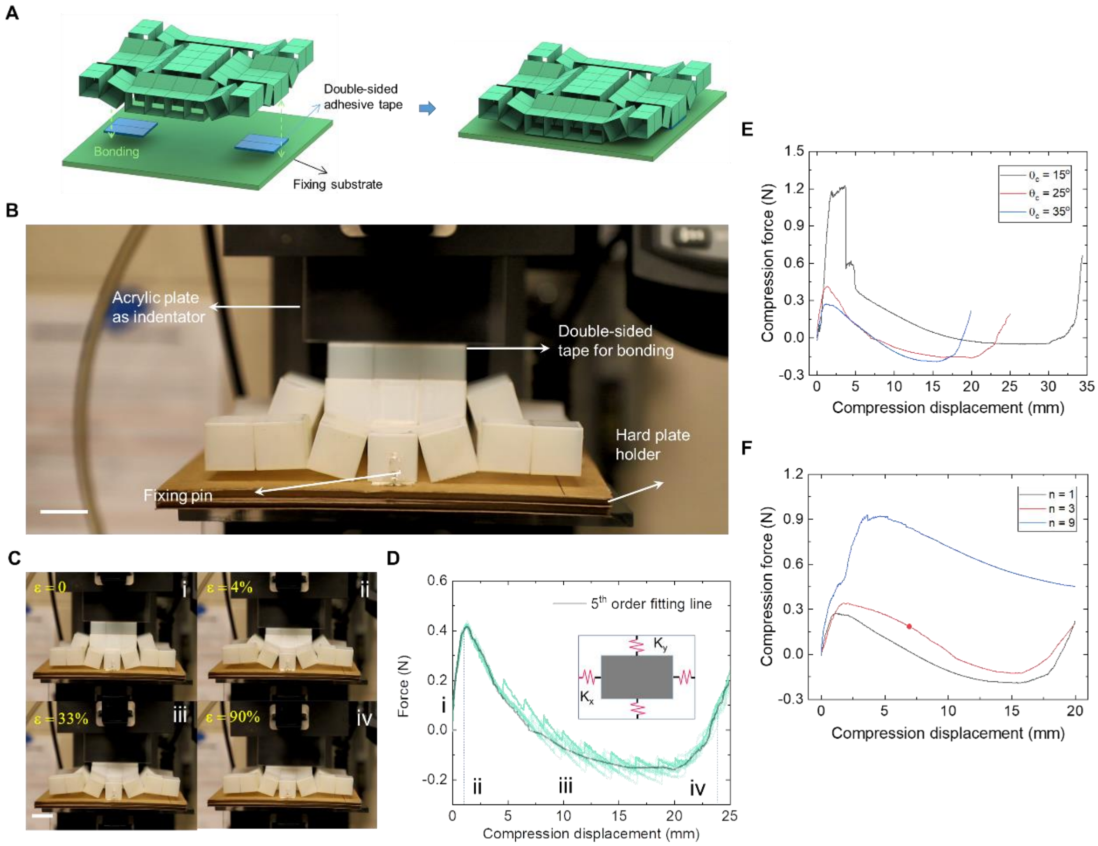

Supplementary Fig. S8. Experimental compression test of the bistable deformation of the unit cell. (**A**) Schematic illustration of the pre-stretched unit with fixing boundaries. (**B**) Front view of the uniaxial compression test of the unit prototype. (**C**) Different configuration state under compression strains as 0, 4%, 33% and 90%. (**D**) The force-displacement curve. (**E** and **F**) Experimental exploration of the structural bistability influenced by pre-stretching strain (equivalently as the center rotation angle θ_c) and hinges stiffness (i.e., the hinge thickness). Scale bar: 2cm.

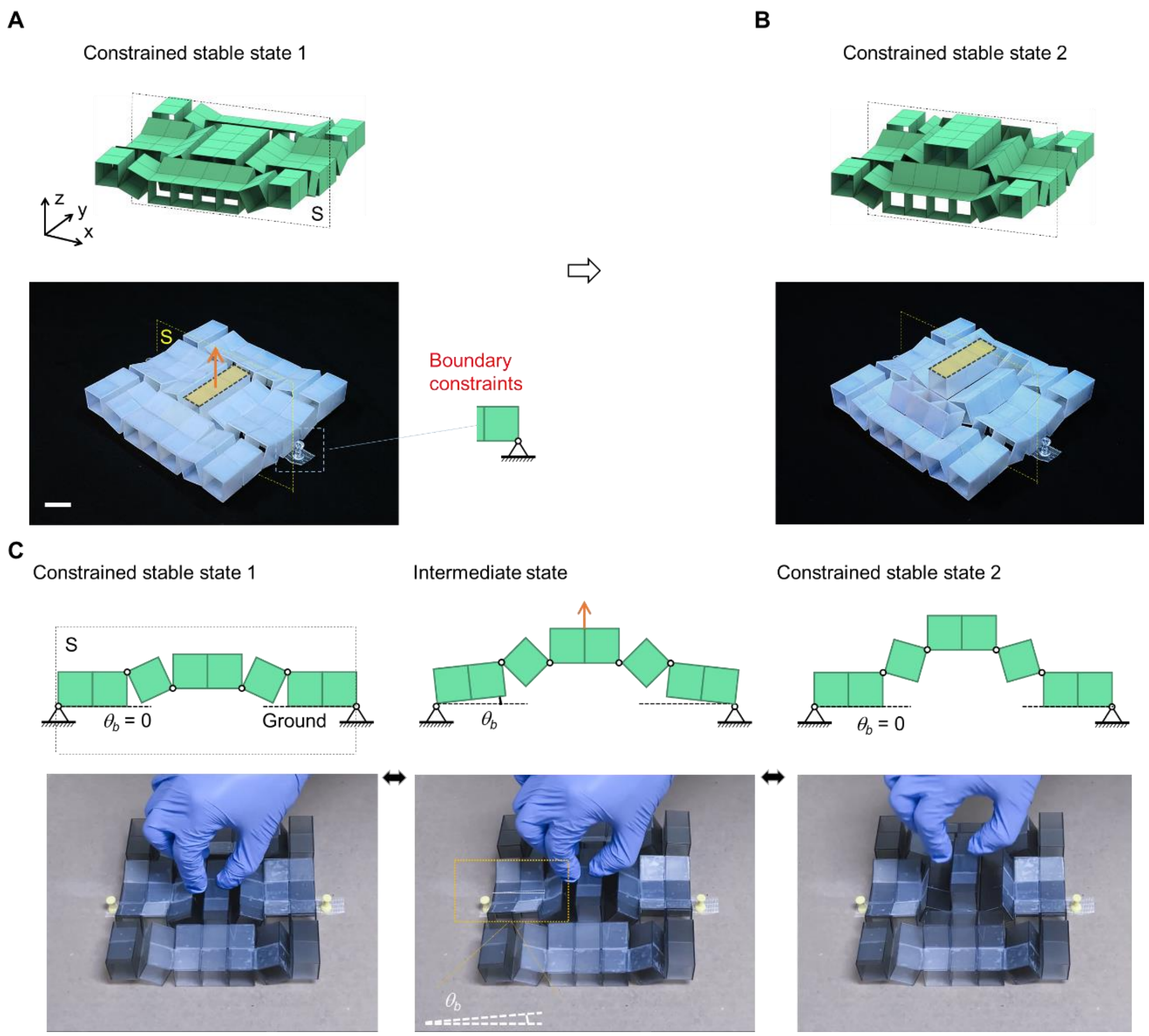

Supplementary Fig. S9 Analysis of the deformed structural profiles of the constrained building block when performing bistable deformations. (**A**) and (**B**) schematics of the first and second constrained stable states. (**C**) Free body diagram and their related experimental images for the deformed structural profile of the constrained building block. Scale bar: 2cm

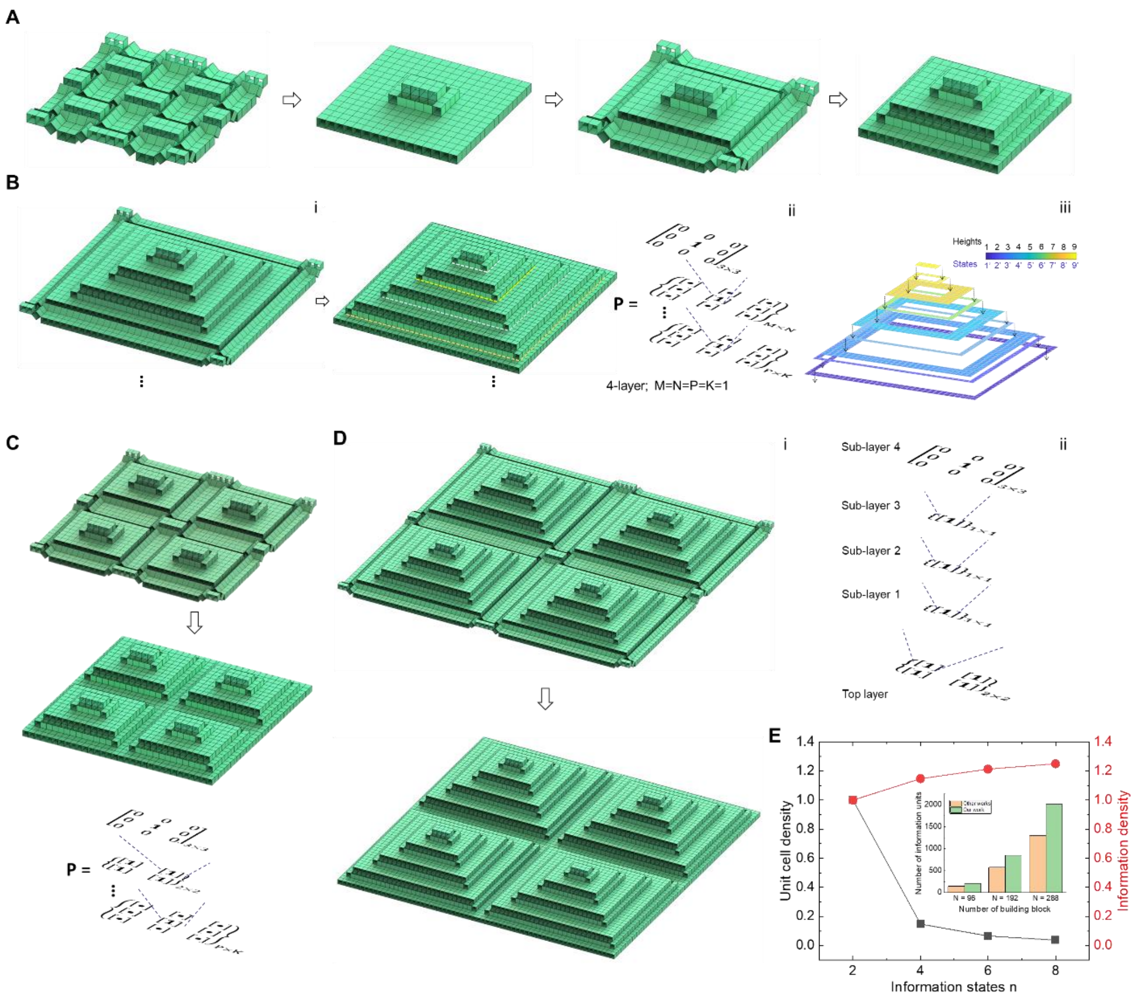

Supplementary Fig. S10 Illustration of the reconfiguration of multilayered configurations. (A) 5 layers. (**B**) 9 layers. (**C**-**D**) Hierarchical assembly of (A) and (B), respectively. (**E**) Variation of the different unit cell density and the increasing information density on a certain mechanical metastructure with respect to their information states n (=2, 4, 6, 8) based on the structure with 96 side cubes. n = 2 (binary), 4 (quaternary), 6 (senary), 8 (octonary).

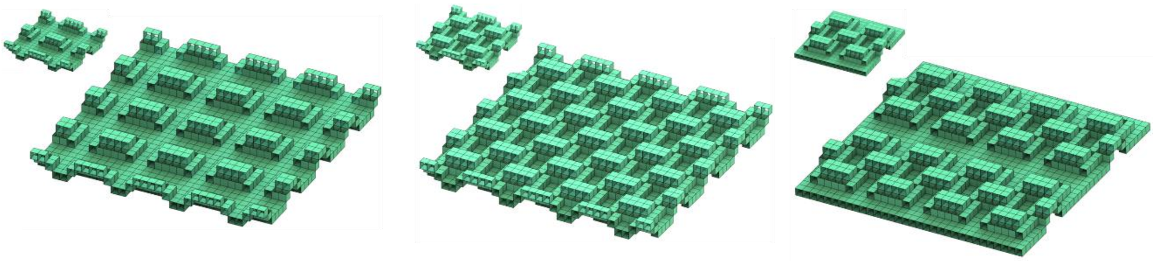

Supplementary Fig. S11. Schematic illustration of the proposed mechanical metastructure reconfigured with periodic surficial morphologies.

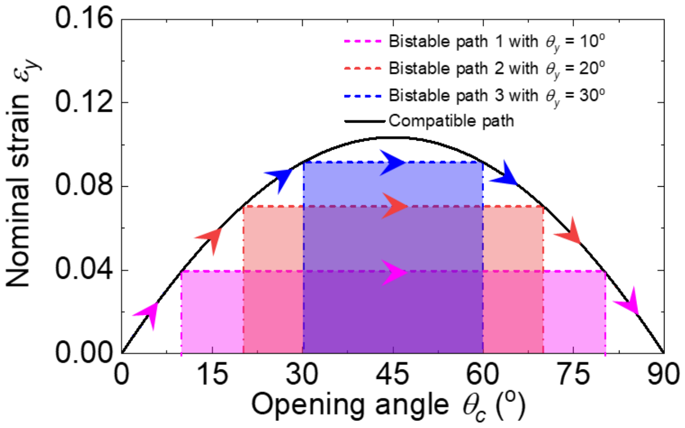

Supplementary Fig. S12. Demonstration of the non-varying length during reconfiguration

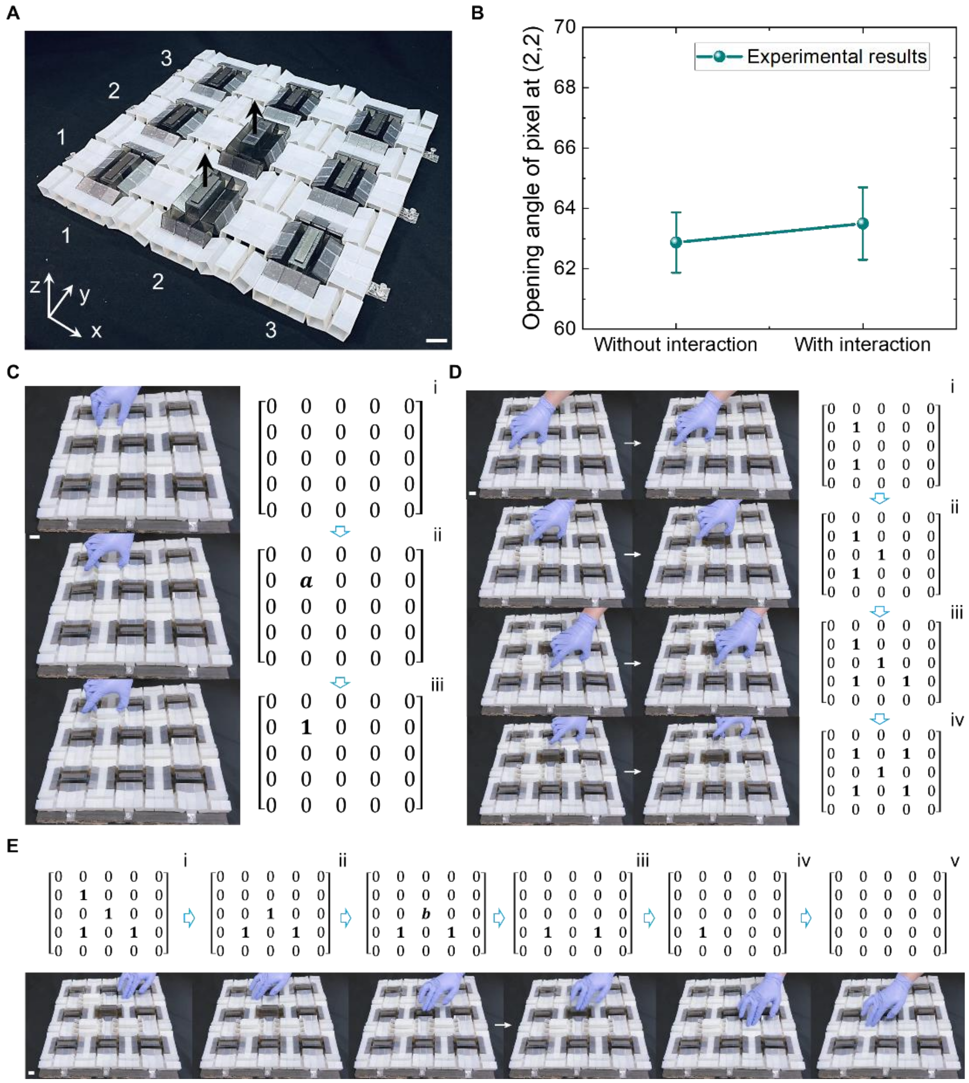

Supplementary Fig. S13. Demonstration of the independent bi-stable deformations among adjacent local structural elements. (**A**) The position of selected two adjacent local structural elements. (**B**) Variation of the opening angle of the central structural elements (2,2) influenced by the repeating bi-stable deformation of the structural element at (2,1). (**C**-**E**) Optical image of the demonstration of (1) programming the deformed pattern without any local support (**C** and **D**) and (2) pulling up (or pushing down) neighboring building blocks without influencing other deformed/non-deformed building blocks (**C** to **E**) on a 3 by 3 tessellated structure. Scale bar: 2cm.

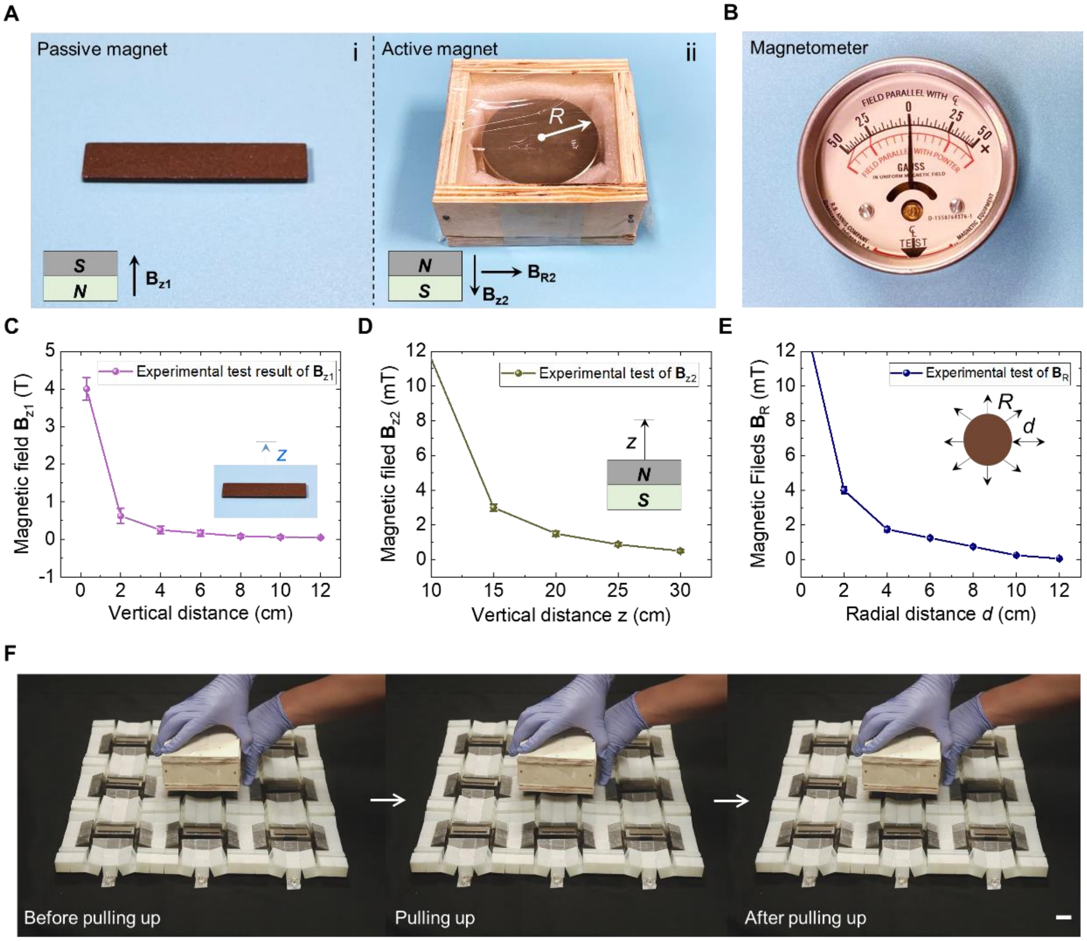

Supplementary Fig. S14. Demonstration of the passive and active permanent magnets used for the untethered bi-stable deformation of local structural elements on mechanical metastructure. (**A**) The used rare-earth passive magnet and active magnet for actuation. (**B**) The used Magnetometer to measure the magnetic field of the passive and active magnets. (**C**) Distribution of the magnetic field $\mathbf{B}_{z1}$ of the passive magnet along its thickness direction. (**D**) Distribution of the magnetic field $\mathbf{B}_{z2}$ of the active magnet along its thickness direction. (**E**) Distribution of the magnetic field $\mathbf{B}_{R}$ of the active magnet along its radial direction. (**F**) Optical image of the magnetic programming process to pull up one local building block. Scale bar: 2cm

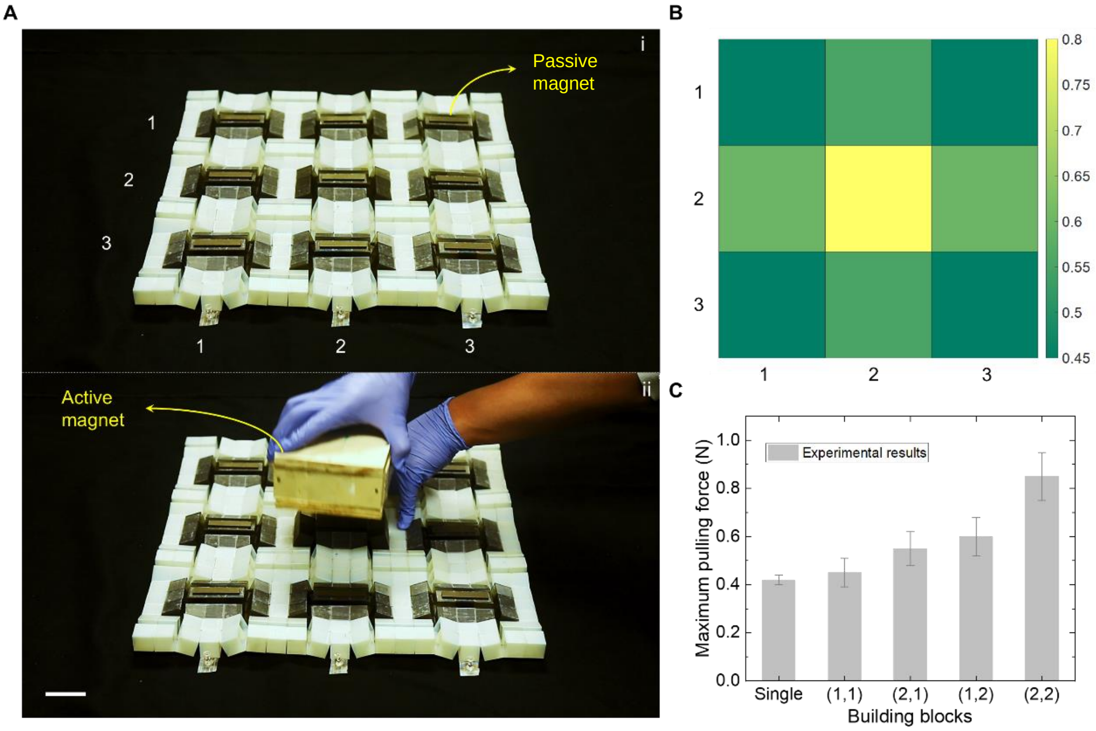

D

i

3 by 3 tessellated structure

(Unit: N)

Position	1	2	3
1	0.45	0.6	0.45
2	0.55	0.85	0.55
3	0.45	0.6	0.45

ii

5 by 5 tessellated structure

(Unit: N)

Position	1	2	3	4	5
1	0.45	0.6	0.62	0.59	0.45
2	0.55	0.83	0.85	0.83	0.55
3	0.57	0.84	0.87	0.84	0.58
4	0.55	0.83	0.85	0.84	0.54
5	0.45	0.6	0.61	0.58	0.45

Supplementary Fig. S15. Pulling force distributions for all the local structural elements of a 3 by 3 mechanical metastructure. (A) Optical images of the metastructure and its actuation. (B-C) Comparison of the measured pulling forces in each single building block. (D) Comparison of maximum pulling force between 3× 3 and 5 × 5 tessellated structure. Scale bar: 4 cm

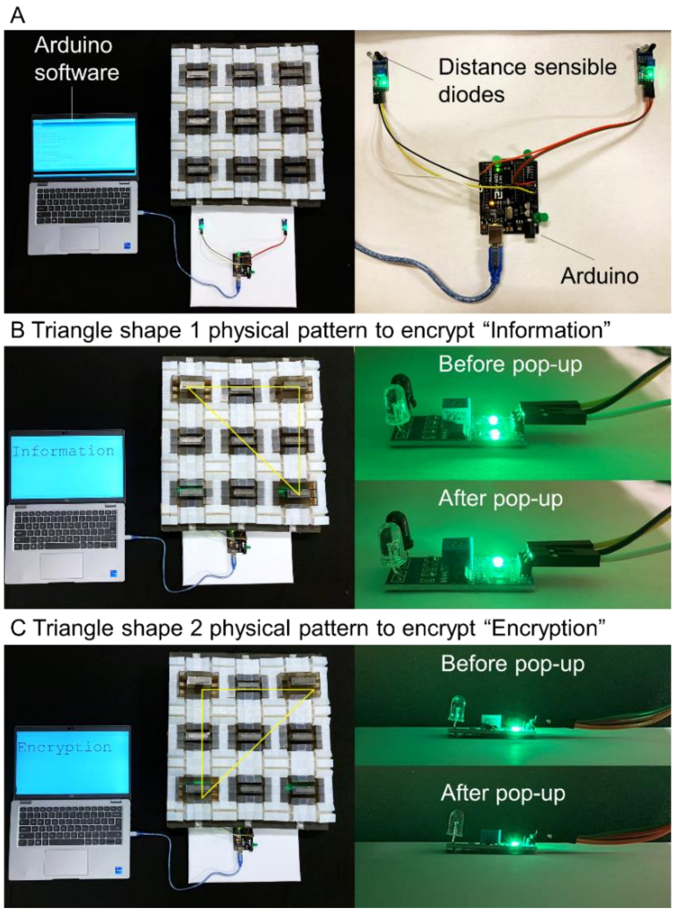

Supplementary Fig. S16. Demonstration of the mechanical metastructure for the structure-based physical information encryption (SPIE). (**A**) Design details of the SPIE system that includes the meta-structure for information encoding, the information transmission system (distance sensible diodes to detect the distance change of local structural element and control board to transmit the physical distance change into electrical signals) and the perception system to percept and display the encoded information. (**B**) Physical demonstration of local structural elements popping up to their second stable states to form a triangle shape that represents the word “Information”. (**C**). Physical demonstration of local structural elements popping up to their second stable states to form a triangle shape that represents the word “Encryption”.

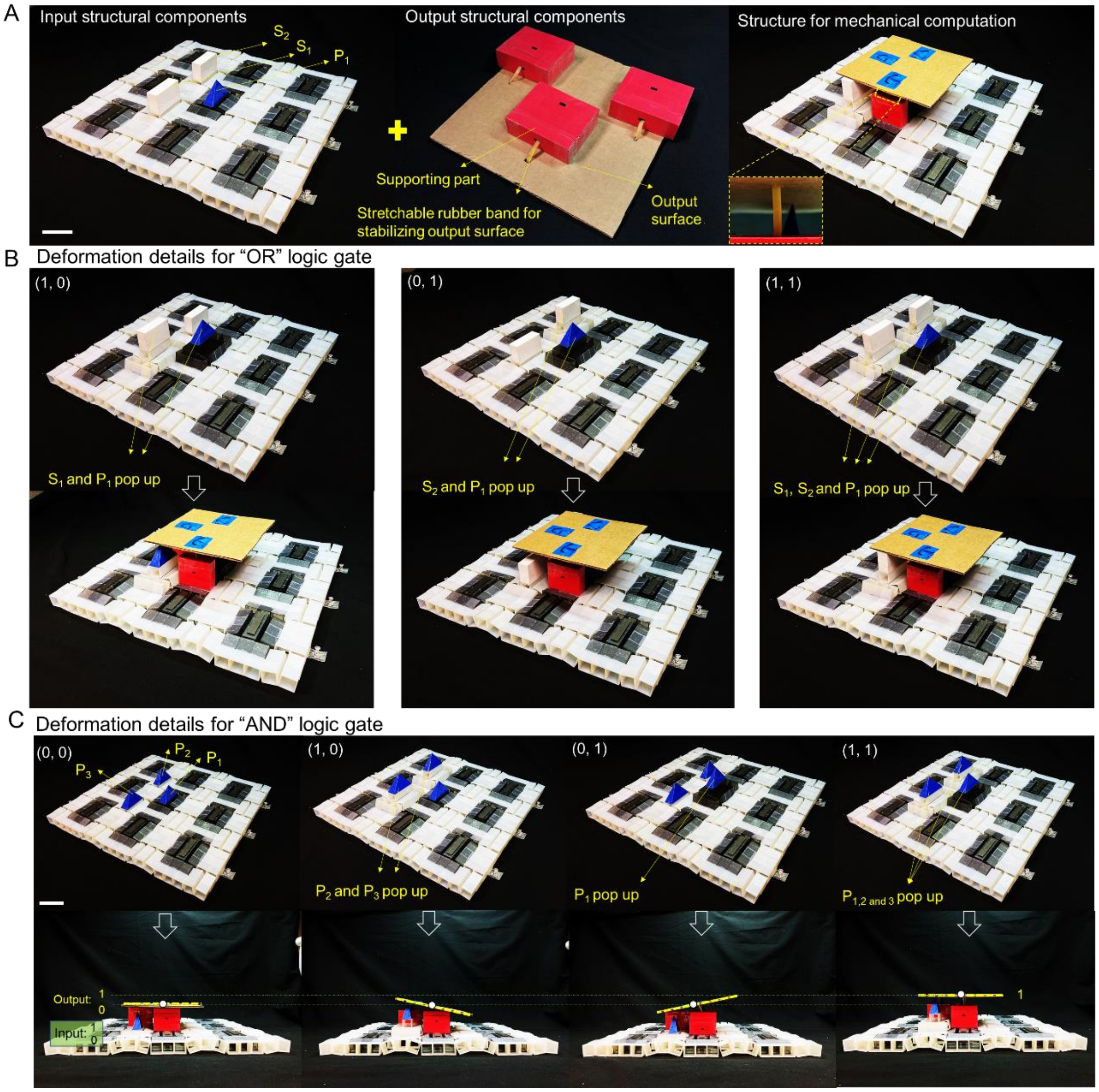

Supplementary Fig. S17. Demonstration of the mechanical metastructure used as mechanical binary logic computations. (**A**) Design details of the additional input and output structural parts. (**B**) Experimental demonstration of the “OR” logic gate. (**C**) Experimental demonstration of the “AND” logic gate. Scale bar: 2 cm.

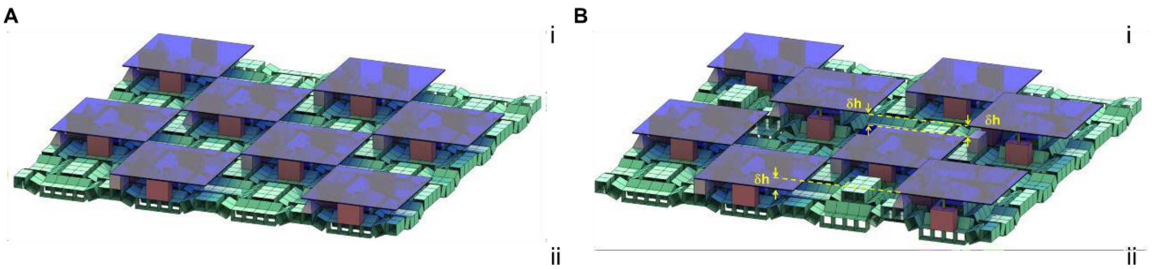

$$\begin{pmatrix} \boldsymbol{and} = \mathbf{0} & null & \boldsymbol{or} = \mathbf{0} & null \\ null & \boldsymbol{and} = \mathbf{0} & null & \boldsymbol{or} = \mathbf{0} \\ \boldsymbol{and} = \mathbf{0} & null & \boldsymbol{or} = \mathbf{0} & null \\ null & \boldsymbol{or} = \mathbf{0} & null & \boldsymbol{and} = \mathbf{0} \end{pmatrix} \qquad \begin{pmatrix} \boldsymbol{and} = \mathbf{0} & null & \boldsymbol{or} = \mathbf{0} & null \\ null & \boldsymbol{and} = \mathbf{1} & null & \boldsymbol{or} = \mathbf{1} \\ \boldsymbol{and} = \mathbf{0} & null & \boldsymbol{or} = \mathbf{0} & null \\ null & \boldsymbol{or} = \mathbf{0} & null & \boldsymbol{and} = \mathbf{1} \end{pmatrix}$$

Supplementary Fig. S18. Demonstration of the mechanical metastructure used as parallel mechanical binary logic computations and their matrix form illustrations. (**A**) Schematics of the mechanical metastructure for parallel mechanical binary logic computations with "***or***" and "***and***" logic value equal to **0**. (**B**) Schematics of independently performing parallel binary mechanical logic computation by selectively setting some local structural elements' logic values equal to "**1**" (see the red color ones in the matrix).

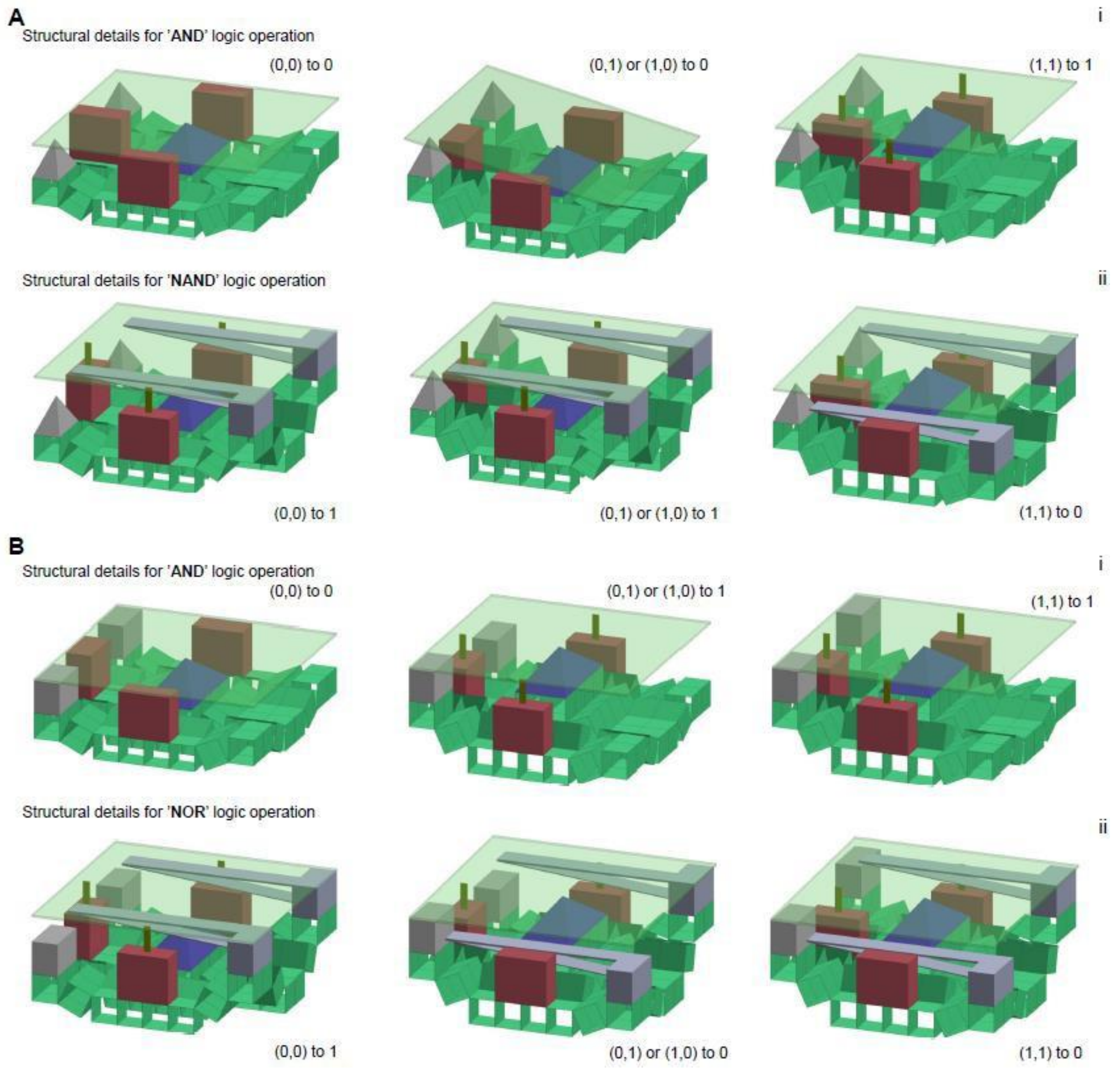

Supplementary Fig. S19. Demonstration of the "NOR" and "NAND" binary logic gate. (**A**) Schematics of the structural details of performing "AND" and "NAND" logic gates on local structural element. (**B**) Schematics of the structural details of performing "AND" and "NOR" logic gates.

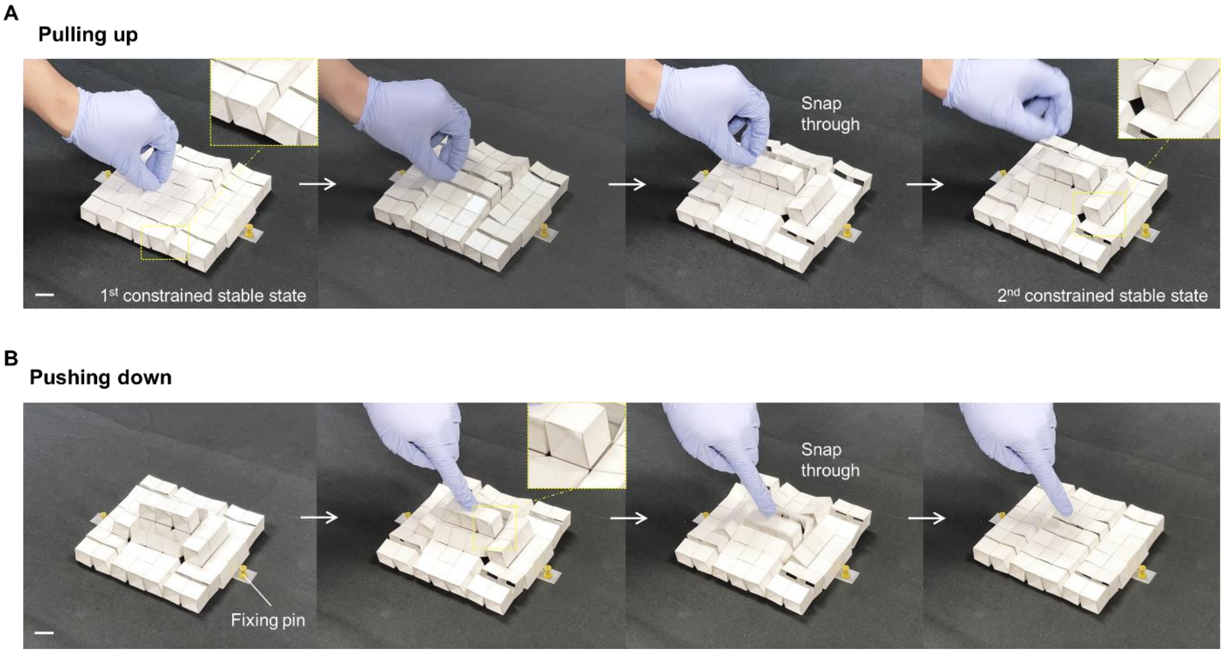

Supplementary Fig. S20 Optical images of the pulling up and pushing down process of building block fabricated by soft cubes. (**A**) Pulling up process of the soft cube fabricated building block with boundary constraints. (**B**) Pushing down process. Scale bar: 2cm

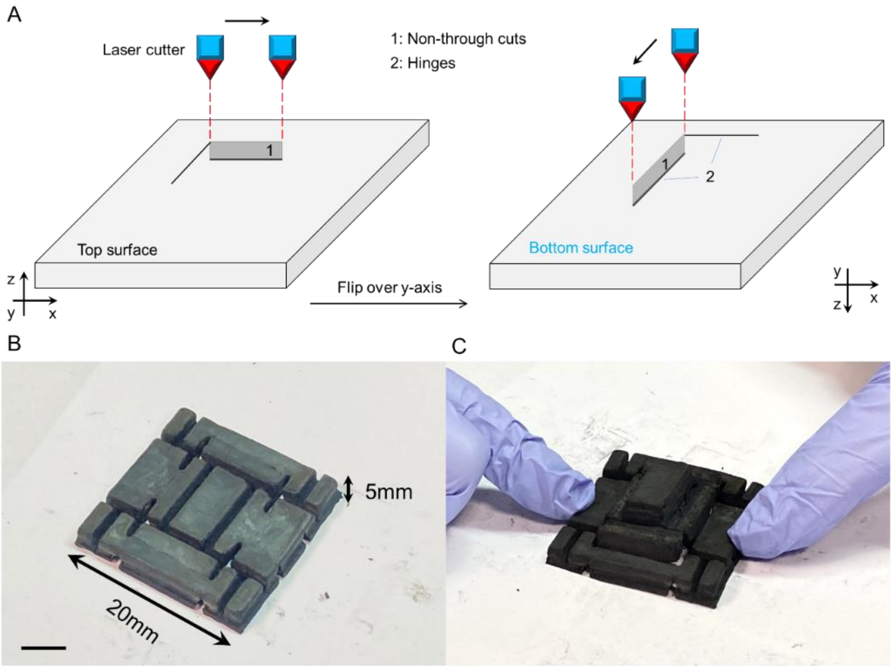

Supplementary Fig. S21. Scalable and one-step fabrication of the mechanical metastructure unit. (**A**) Schematic illustration of the simple fabrication method of our built mechanical metastructrue through directly introducing non-through slits onto thick panel solid. (**B**) Unit (2cm by 2cm by 0.5cm) prototype based on thick rubber panel with only laser-cutting technique. (**C**) Demonstration of the structural bi-stability. Scale bar: 5mm.

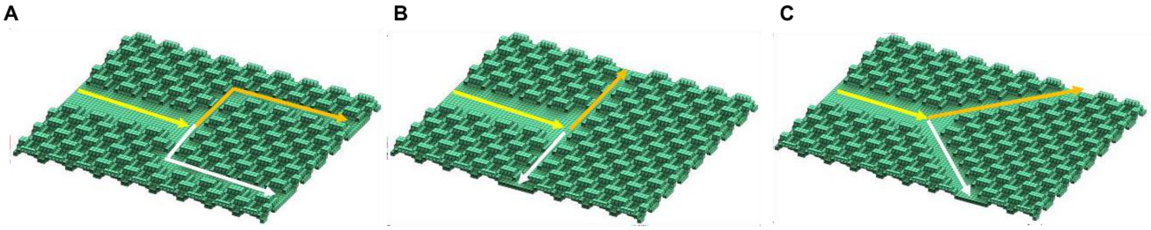

Supplementary Fig. S22. Demonstration of the mechanical metastructure with other application platform with surficial channels. (**A**) Connected "I"+"U" shape channel. (B) "T" shape channel. (**C**) "Y" shape channel.

Supplementary Movies

Movie S1: Reconfiguration details of level-1 and level-2 building blocks

Movie S2: Reconfiguration details of structural unit

Movie S3: Bi-stability deformation of structural unit

Movie S4: Reconfigurations of periodic structure

Movie S5: Magnetic actuation demonstration for the bistability of structural unit

Movie S6: Application for mechanical memory storage device actuated with magnet

Movie S7: Application for physical information interaction actuated with magnet

Movie S8: Application for physical information encryption

Movie S9: Extension to other surface system using thick rubber plate
